# Comparative Study of Molecular Descriptors of Pent-Heptagonal Nanostructures Using Neighborhood M-Polynomial Approach

**DOI:** 10.3390/molecules28062518

**Published:** 2023-03-09

**Authors:** D. Antony Xavier, Muhammad Usman Ghani, Muhammad Imran, Theertha Nair A., Eddith Sarah Varghese, Annmaria Baby

**Affiliations:** 1 Department of Mathematics, Loyola College, Chennai 600034, India; 2 Institute of Mathematics, Khawaja Fareed University of Engineering & Information Technology, Abu Dhabi Road, Rahim Yar Khan 64200, Pakistan; 3 Department of Mathematical Sciences, United Arab Emirates University, Al Ain P.O. Box 15551, United Arab Emirates

**Keywords:** pent-heptagonal carbon sheets, M-polynomial, neighborhood degree, molecular graphs, entropy, regression analysis

## Abstract

In this article, a novel technique to evaluate and compare the neighborhood degree molecular descriptors of two variations of the carbon nanosheet $C_{5}C_{7}\left(a,b\right)$ is presented. The conjugated molecules follow the graph spectral theory, in terms of bonding, non-bonding and antibonding Ruckel molecular orbitals. They are demonstrated to be immediately determinable from their topological characteristics. The effort of chemical and pharmaceutical researchers is significantly increased by the need to conduct numerous chemical experiments to ascertain the chemical characteristics of such a wide variety of novel chemicals. In order to generate novel cellular imaging techniques and to accomplish the regulation of certain cellular mechanisms, scientists have utilized the attributes of nanosheets such as their flexibility and simplicity of modification, out of which carbon nanosheets stand out for their remarkable strength, chemical stability, and electrical conductivity. With efficient tools like polynomials and functions that can forecast compound features, mathematical chemistry has a lot to offer. One such approach is the M-polynomial, a fundamental polynomial that can generate a significant number of degree-based topological indices. Among them, the neighborhood M-polynomial is useful in retrieving neighborhood degree sum-based topological indices that can help in carrying out physical, chemical, and biological experiments. This paper formulates the unique M-polynomial approach which is used to derive and compare a variety of neighborhood degree-based molecular descriptors and the corresponding entropy measures of two variations of pent-heptagonal carbon nanosheets. Furthermore, a regression analysis on these descriptors has also been carried out which can further help in the prediction of various properties of the molecule.

## 1. Introduction

The rising number of technologies and applications for nanoscience has sparked scientific interest. With a thickness ranging from 1 to 100 nm, a nanosheet is a two-dimensional nanostructure. A significant number of novel nanomaterials, crystalline materials and pharmaceuticals are developed every year as a result of the rapid evolution of chemical and pharmaceutical processes [1]. Nanotechnology has a significant and positive impact on the situation. The utilization of nanomaterials and nano-objects in many chemical, biological and technical domains are widely preferred. Carbon nanotubes are one of the most popular nanostructures.

Carbon nanotubes are graphitic carbon tubes that are made at the molecular scale and have exceptional characteristics. They exhibit impressive electrical capabilities and are among the stiffest and strongest fibers ever discovered. Due to these factors, a significant number of articles are produced every year, attracting both academic and commercial attention [2]. Carbon nanosheets (CNSs) with thicknesses on the nanoscale range can be used for a variety of applications, including biosensors, field electron emissions, batteries, fuel cells, hydrogen-storage materials, catalyst supports and ultracapacitor electrodes. This is because CNSs have an extremely high surface-to-volume ratio. Moreover, it has been claimed that 2D carbon nanostructures are a very sensitive gas-detecting material [3]. Many of the applications involving these nanostructures can be found in [4,5].

Any graph that models a chemical structure can be described mathematically using a topological graph descriptor [6,7,8,9]. From this index value, it is possible to analyze mathematical values and further investigate various physico-chemical properties of a molecule [10]. As a result, it is an effective way to eliminate expensive and time-consuming experimental research. With powerful tools like polynomials and functions that can forecast the chemical features of a molecule, mathematical chemistry has a lot to offer. One of the greatest advancements in this field is the development of the M-polynomial concept [11,12]. It is tedious to compute topological indices using their standard definitions in many cases [13,14,15]. Hence to overcome this strategy, numerous algebraic polynomials have been developed such that the differentiation, integration or composition of both of which are determined at a given point, can yield a variety of topological indices [16,17]. The polynomial that produces the highest number of degree-based topological indices is the M-polynomial [18]. Recently, more amount of research has been carried out based on neighborhood degree indices. The neighborhood M-polynomial is useful for obtaining neighborhood degree sum-based molecular descriptors that can forecast various physical, chemical and biological properties of the material under consideration.

**Figure 1:**
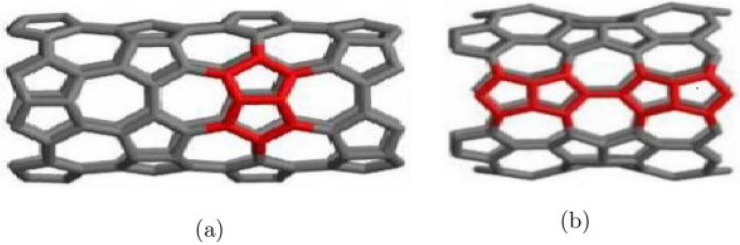
(**a**) Molecular graph of $VC_{5}C_{7}$, (**b**) Molecular graph of $HC_{5}C_{7}$.

In the context of M-polynomial, a good amount of study has been conducted, as in the case of Munir et al., who processed M-polynomial and associated lists of triangular boron nanotubes, polyhex nanotubes, nanostar dendrimers and titania nanotubes to name a few [19,20,21]. Numerous studies on the neighborhood degree-based topological index have also been done in recent times. In this research, two Pent heptagonal carbon nanosheet variants have been investigated whose molecular graphs are shown in Figure 1. A great amount of research has been invested in these nanosheets, including the degree, distance and a few neighborhood degree-based molecular descriptors, the M-polynomial degree approach, the development of different polynomials like Zagreb polynomials, the evaluation of irregularity index, connectivity index and many more [22,23,24,25,26,27]. The M-polynomial approach to the neighborhood degree for these nanosheets remains a research gap. This study fills the gap in the current framework since the neighborhood degree descriptors and M-polynomial function have several applications, including the measurement of the acentric factor, the calculation of enthalpy and the determination of heat capacity to mention a few [28]. Additionally, this work has been expanded by applying Shannon’s entropy model to calculate the Entropy using these descriptors [29,30,31]. Moreover, a comparison of the two variants of these nanosheets has also been carried out using various graphing tools.

## 2. Chemical Significance of Molecular Descriptors

Any graph that mimics a particular molecular structure can be given a topological graph index, also known as a molecular descriptor [27]. From this index, it is possible to analyze numerical numbers and further look into some of the molecule’s physical characteristics. As a result, it is a useful technique to eliminate costly and time-consuming laboratory studies. In mathematical chemistry, molecular descriptors are crucial, particularly in studies of quantitative structure-property relationships (QSPR) and quantitative structure-activity relationships (QSAR) [28]. Drug design in medical research depends on the chemical, physiological, biological and pharmacological aspects of molecular structure. Different mathematical tools are used to forecast certain chemistries’ features, such as topological index, entropy and enthalpy to name a few. The topological index allows us to link a single number to a molecular graph of a chemical complex. Polygonal forms, trees, graphs, and other geometrical shapes are widely used to represent drugs and other chemical compounds. In this study, we discuss the newly introduced neighborhood version of the invariants for the pent-heptagonal nanostructures (third Zagreb index, second Zagreb index, *F*-index, general Randic index, second modified Zagreb, inverse sum index, harmonic index and Sanskruti index). The goal of this study is to give the reader a current overview of the novel descriptors of pent-heptagonal nanostructures which can help further in the analysis of chemical properties. Some of the chemical attributes which can be predicted using these indices include boiling point (BP), enthalpy *S*, acentric factor ω, enthalpy of formation $\Delta H_{f}$, octanol-water partition coefficient (log *P*), Kovats retention index (RI). With proper statistical analysis using regression and proper correlation, these descriptors can predict various physico-chemical properties of compounds [32].

Like, Ghani at all. in [31] start works on entropy by using topological indices. A topological descriptor is an illustration of a molecular descriptor. There are several topological indices available today, some of which are used in chemistry [8,20]. The structural characteristics of the graphs utilized for their computation can be used to categorize them. The Hosoya index, for instance, is determined by counting non-incident edges in a graph. In addition, the degrees of vertices are used to generate the Randic connectivity index, the Zagreb group indices, the Estrada index and other indices. Different innovative approaches have also been established for usage in QSPR analysis such as the electrotopological state index, which incorporates both the electronic character and the topological surroundings of each skeletal atom in a molecule to characterize atoms in molecules. In this approach, the structure is represented by the hydrogen-suppressed graph. Examples of numerous organic framework types such as chain lengthening, branching, heteroatoms and unsaturation, serve as indicators for the properties of the electron topological state values [33,34]. Certain other insightful studies have also been conducted, such as virtual screening, which uses a few basic characteristics, such as the number of atoms in each element, to describe molecules without taking into account their structural constitution [35]. Further discussion on molecular branching and other attributes can be seen in [36,37].

## 3. Mathematical Terminologies

**Figure 2:**
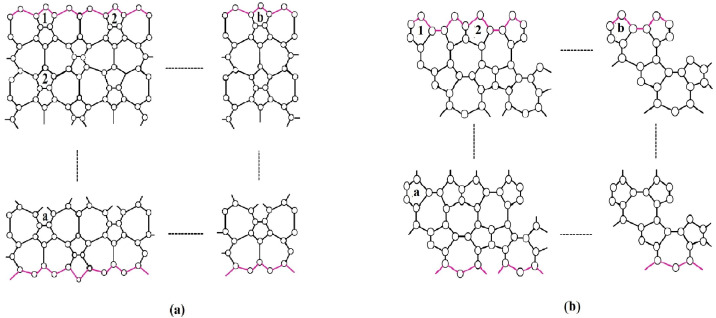
(**a**) 2-D lattice of $VC_{5}C_{7}\left(a,b\right)$, (**b**) 2-D lattice of $HC_{5}C_{7}\left(a,b\right)$.

We first explain the growth pattern of the nanosheets. The 2-dimensional lattice of the nanosheets $VC_{5}C_{7}\left(a,b\right)$ and $HC_{5}C_{7}\left(a,b\right)$ is shown in Figure 2. Here, for both structures, *b* denotes the number of pentagons in the first row and *a* represents the number of repetitions. On observing and generalizing the pattern of growth, we obtain that $VC_{5}C_{7}\left(a,b\right)$ contains 16ab+2a+5b vertices and 24ab+4b edges whereas $HC_{5}C_{7}\left(a,b\right)$ contains 16ab+2a+4b vertices and 24ab+3b edges.

The neighborhood degree of a vertex is defined to be the sum of the degrees of its adjacent vertices. The concept which is used here is neighborhood degree M-polynomial and is denoted by NM(Γ;x,y) for a simple connected graph Γ and is defined to be

(1) $$NM\left(\Gamma ;x,y\right)=\sum_{u\leq v}m_{\left(u,v\right)}x^{u}y^{v}$$

where $m_{\left(u,v\right)}$ is the total number of edges ef∈E(Γ) such that $\left\{\delta_{e},\delta_{f}\right\}=\left\{u,v\right\}$. Throughout the paper, we use the notation NM(Γ) for NM(Γ;x,y).

In general, as defined on the set of a graph Γ, the neighborhood degree-based topological indices can be written as;

$$I\left(\Gamma \right)=\sum_{ef\in E(\Gamma )}f\left(\delta_{e},\delta_{f}\right).$$

where $f(\delta_{e},\delta_{f})$ is the function used in defining of neighborhood degree descriptors. This outcome can therefore be expressed as,

(2) $$I\left(\Gamma \right)=\sum_{u\leq v}m_{\left(u,v\right)}f\left(u,v\right).$$

Some of the fundamental research works involved in the building of this concept can be seen in [11,38,39,40,41]. Now, we discuss various neighborhood degree descriptors.

The third version of the Zagreb index is expressed to be,

$$M_{1}^{\prime }\left(\Gamma \right)=\sum_{ef\in E(\Gamma )}\left(\delta_{e}+\delta_{f}\right).$$

The neighborhood second Zagreb index is expressed to be,

$$M_{2}^{*}\left(\Gamma \right)=\sum_{ef\in E(\Gamma )}\delta_{e}\delta_{f}.$$

The neighborhood forgotten topological index is expressed to be,

$$F_{N}^{*}\left(\Gamma \right)=\sum_{ef\in E(\Gamma )}\left(\delta_{e}^{2}+\delta_{f}^{2}\right).$$

The neighborhood second modified Zagreb index is expressed to be,

$$M_{2}^{nm}\left(\Gamma \right)=\sum_{ef\in E(\Gamma )}\frac{1}{\delta_{e}\delta_{f}}.$$

The neighborhood general Randic index is expressed to be,

$$NR_{\alpha }\left(\Gamma \right)=\sum_{ef\in E(\Gamma )}{\left(\delta_{e}\delta_{f}\right)}^{\alpha }.$$

The third NDe index is expressed to be,

$$ND_{3}\left(\Gamma \right)=\sum_{ef\in E(\Gamma )}\delta_{e}\delta_{f}\left(\delta_{e}+\delta_{f}\right).$$

The fifth NDe index is expressed to be,

$$ND_{5}\left(\Gamma \right)=\sum_{ef\in E(\Gamma )}\left[\frac{\delta_{e}}{\delta_{f}}+\frac{\delta_{e}}{\delta_{f}}\right].$$

The neighborhood Harmonic index is expressed to be,

$$NH\left(\Gamma \right)=\sum_{ef\in E(\Gamma )}\frac{2}{\delta_{e}+\delta_{f}}.$$

The neighborhood inverse sum index is expressed to be,

$$NI\left(\Gamma \right)=\sum_{ef\in E(\Gamma )}\frac{\delta_{e}\delta_{f}}{\delta_{e}+\delta_{f}}.$$

The Sanskruti index is expressed to be,

$$S\left(\Gamma \right)=\sum_{ef\in E(\Gamma )}{\left(\frac{\delta_{e}\delta_{f}}{\delta_{e}+\delta_{f}-2}\right)}^{3}.$$

Table 1 displays the correlations between the NM-polynomial and a few neighborhood degree-based topological indices.

Here,

$$D_{x}\left(f\left(x,y\right)\right)=x\frac{\partial (f(x,y))}{\partial x},D_{y}\left(f\left(x,y\right)\right)=y\frac{\partial (f(x,y))}{\partial y},S_{x}\left(f\left(x,y\right)\right)=\int_{0}^{x}\frac{f(t,y)}{t}dt,$$

$$S_{y}\left(f\left(x,y\right)\right)=\int_{0}^{y}\frac{f(x,t)}{t}dt,J\left(f\left(x,y\right)\right)=f\left(x,x\right),Q_{\alpha }\left(f\left(x,y\right)\right)=x^{\alpha }f\left(x,y\right).$$

**Figure 3:**
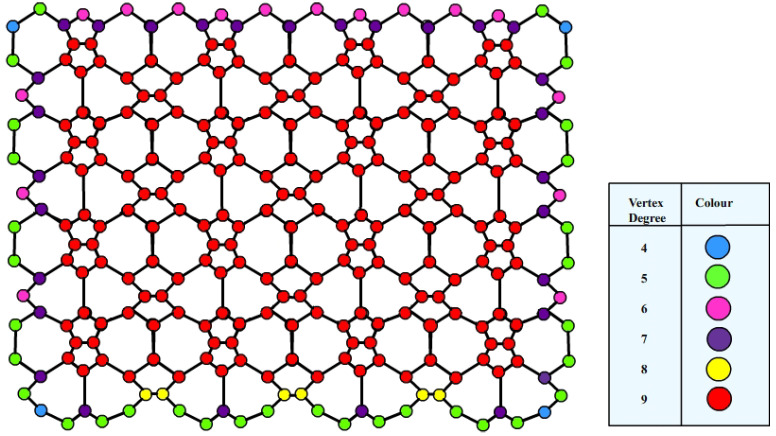
Neighborhood degree illustration of $VC_{5}C_{7}\left[4,4\right]$.

**Figure 4:**
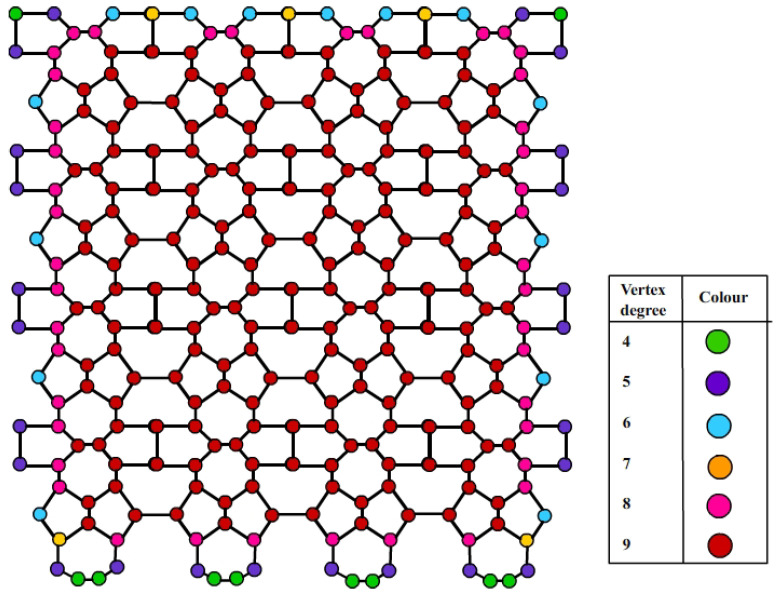
Neighborhood degree illustration of $HC_{5}C_{7}\left[4,4\right]$.

A diagrammatic representation of neighborhood degrees of nanosheets $VC_{5}C_{7}\left[4,4\right]$ and $HC_{5}C_{7}\left[4,4\right]$ are shown in Figure 3 and Figure 4, respectively.

**Theorem** **1.** *Let* Γ *be a graph of carbon nanosheet $VC_{5}C_{7}\left[a,b\right]$, then we have $NM\left(VC_{5}C_{7}\left[a,b\right]\right)=\left(24ab-15b-14a+10\right)x^{9}y^{9}+\left(2b-2\right)x^{8}y^{9}+\left(b-1\right)x^{8}y^{8}+\left(4a+4b-3\right)x^{7}y^{9}+\left(4a+6b-8\right)x^{6}y^{7}+\left(2b-2\right)x^{5}y^{8}+\left(4a+2b+2\right)x^{5}y^{7}+\left(2a+2b-4\right)x^{5}y^{5}+8x^{4}y^{5}.$*

**Proof.**  By analyzing the construction of $VC_{5}C_{7}\left[a,b\right]$, the graph has 16ab+2a+5b vertices and 24ab+4b edges. The neighborhood degree of vertices of $VC_{5}C_{7}\left[a,b\right]$ can be 4,5,6,7,8 or 9 and hence the 9 partitions for the edge set based on the neighborhood degree are (4,5), (5,5), (5,7), (5,8), (6,7), (7,9), (8,8), (8,9) and (9,9). The edge partitions $\left(d_{x},d_{y}\right)$, $xy\in E(VC_{5}C_{7}\left[a,b\right])$ and the corresponding number of edges in each partition is given in Table 2.

From the definition of NM-polynomial of $VC_{5}C_{7}$ is obtained as,

$$\begin{array}{cc} NM(VC_{5}C_{7};x,y)= & \sum_{i\leq j}m_{\left(i,j\right)}x^{i}y^{j}\\ = & m_{\left(4,5\right)}x^{4}y^{5}+m_{\left(5,5\right)}x^{5}y^{5}+m_{\left(5,7\right)}x^{5}y^{7}+m_{\left(5,8\right)}x^{5}y^{8}+m_{\left(6,7\right)}x^{6}y^{7}+m_{\left(7,9\right)}x^{7}y^{9}\\ + & m_{\left(8,8\right)}x^{8}y^{8}+m_{\left(8,9\right)}x^{8}y^{9}+m_{\left(9,9\right)}x^{9}y^{9}\\ = & 8x^{4}y^{5}+\left(2b+2a-4\right)x^{5}y^{5}+\left(2b+4a+2\right)x^{5}y^{7}+\left(2b-2\right)x^{5}y^{8}\\ + & \left(6b+4a-8\right)x^{6}y^{7}+\left(4b+4a-3\right)x^{7}y^{9}+\left(24ab-15b-14a+10\right)x^{9}y^{9}\\ + & \left(b-1\right)x^{8}y^{8}+\left(2b-2\right)x^{8}y^{9}\\ = & \left(24ab-15b-14a+10\right)x^{9}y^{9}+\left(2b-2\right)x^{8}y^{9}+\left(b-1\right)x^{8}y^{8}\\ + & \left(4a+4b-3\right)x^{7}y^{9}+\left(4a+6b-8\right)x^{6}y^{7}+\left(2b-2\right)x^{5}y^{8}\\ + & \left(4a+2b+2\right)x^{5}y^{7}+\left(2a+2b-4\right)x^{5}y^{5}+8x^{4}y^{5}. \end{array}$$

□

Now using this NM-polynomial, some neighborhood degree-based structural descriptors of $VC_{5}C_{7}$ is obtained.

**Theorem** **2.** *Let* Γ *be $VC_{5}C_{7}\left[a,b\right]$, then*

*1.*  $M_{1}^{\prime }\left(\Gamma \right)=432ab-8b-68a+8$
*2.*  $M_{2}^{nm}\left(\Gamma \right)=\frac{2554a}{14175}+\frac{76117b}{302400}+\frac{8ab}{27}+\frac{80833}{907200}$
*3.*  $NR_{\alpha }\left(\Gamma \right)=35^{\alpha }\left(4a+2b+2\right)+42^{\alpha }\left(4a+6b-8\right)+63^{\alpha }\left(4a+4b-3\right)+2^{6\alpha }\left(b-1\right)+40^{\alpha }\left(2b-2\right)+72^{\alpha }\left(2b-2\right)+5^{2\alpha }\left(2a+2b-4\right)-3^{4\alpha }\left(14a+15b-24ab-10\right)+2^{2\alpha +3}5^{\alpha }$
*4.*  $ND_{3}\left(\Gamma \right)=34992ab-8710b-12016a+3956$
*5.*  $ND_{5}\left(\Gamma \right)=\frac{254a}{315}+\frac{1147b}{126}+48ab-\frac{29}{126}$
*6.*  $NH\left(\Gamma \right)=\frac{733a}{1170}+\frac{30703b}{26520}+\frac{8ab}{3}+\frac{2953}{19890}$
*7.*  $NI\left(\Gamma \right)=108ab-\frac{7711b}{2652}-\frac{2755a}{156}+\frac{5699}{2448}$

**Proof.**  Let $\Gamma =VC_{5}C_{7}$. Using the edge partition table given in Table 2, and using the expressions given in Table 1, various neighborhood degree based structural descriptors are obtained as follows;

We have;
  $NM\left(\Gamma ;x,y\right)=\left(24ab-15b-14a+10\right)x^{9}y^{9}+\left(2b-2\right)x^{8}y^{9}+\left(b-1\right)x^{8}y^{8}+\left(4a+4b-3\right)x^{7}y^{9}+\left(4a+6b-8\right)x^{6}y^{7}+\left(2b-2\right)x^{5}y^{8}+\left(4a+2b+2\right)x^{5}y^{7}+\left(2a+2b-4\right)x^{5}y^{5}+8x^{4}y^{5}.$
  Then,

1. $\left(D_{x}+D_{y}\right)\left(NM\left(\Gamma \right)\right)=40x^{4}y^{4}+x\left(32x^{3}y^{5}-9x^{8}y^{9}\left(14a+15b-24ab-10\right)+5x^{4}y^{8}\left(2b-2\right)+8x^{7}y^{9}\left(2b-2\right)+8x^{7}y^{8}\left(b-1\right)+5x^{4}y^{5}\left(2a+2b-4\right)+5x^{4}y^{7}\left(4a+2b+2\right)+7x^{6}y^{9}\left(4a+4b-3\right)+6x^{5}y^{7}\left(4a+6b-8\right)\right)-9x^{9}y^{8}\left(14a+15b-24ab-10\right)+8x^{5}y^{7}\left(2b-2\right)+9x^{8}y^{8}\left(2b-2\right)+8x^{8}y^{7}\left(b-1\right)+5x^{5}y^{4}\left(2a+2b-4\right)+7x^{5}y^{6}\left(4a+2b+2\right)+9x^{7}y^{8}\left(4a+4b-3\right)+7x^{6}y^{6}\left(4a+6b-8\right)$
  At x=1,y=1;
  $M_{1}^{\prime }\left(VC_{5}C_{7}\right)=432ab-8b-68a+8$
2. $\left(S_{x}S_{y}\right)\left(NM\left(\Gamma \right)\right)=\frac{2x^{4}y^{5}}{5}-\frac{x^{9}y^{9}\left(14a+15b-24ab-10\right)}{81}+\frac{x^{5}y^{8}\left(2b-2\right)}{40}+\frac{x^{8}y^{9}\left(2b-2\right)}{72}+\frac{x^{8}y^{8}\left(b-1\right)}{64}+\frac{x^{5}y^{5}\left(2a+2b-4\right)}{25}+\frac{x^{5}y^{7}\left(4a+2b+2\right)}{35}+\frac{x^{7}y^{9}\left(4a+4b-3\right)}{63}+\frac{x^{6}y^{7}\left(4a+6b-8\right)}{42}$
  At x=1,y=1;
  $M_{2}^{nm}\left(VC_{5}C_{7}\right)=\frac{2554a}{14175}+\frac{76117b}{302400}+\frac{8ab}{27}+\frac{80833}{907200}$
3. $\left(D_{x}^{\alpha }+D_{y}^{\alpha }\right)\left(NM\left(\Gamma \right)\right)=-9^{2\alpha }\left(14a+15b-24ab-10\right)x^{9}y^{9}+8^{\alpha }9^{\alpha }\left(2b-2\right)x^{8}y^{9}+8^{2\alpha }\left(b-1\right)x^{8}y^{8}+7^{\alpha }9^{\alpha }\left(4a+4b-3\right)x^{7}y^{9}+6^{\alpha }7^{\alpha }\left(4a+6b-8\right)x^{6}y^{7}+5^{\alpha }8^{\alpha }\left(2b-2\right)x^{5}y^{8}+5^{\alpha }7^{\alpha }\left(4a+2b+2\right)x^{5}y^{7}+5^{2\alpha }\left(2a+2b-4\right)x^{5}y^{5}+4^{\alpha }5^{\alpha }8x^{4}y^{5}.$
  At x=1,y=1;
  $NR_{\alpha }\left(\Gamma \right)=35^{\alpha }\left(4a+2b+2\right)+42^{\alpha }\left(4a+6b-8\right)+63^{\alpha }\left(4a+4b-3\right)+2^{6\alpha }\left(b-1\right)+40^{\alpha }\left(2b-2\right)+72^{\alpha }\left(2b-2\right)+5^{2\alpha }\left(2a+2b-4\right)-3^{4\alpha }\left(14a+15b-24ab-10\right)+2^{2\alpha +3}5^{\alpha }$
4. $D_{x}D_{y}\left(D_{x}+D_{y}\right)\left(NM\left(\Gamma \right)\right)=2x^{4}y^{5}\left(7290x^{5}y^{4}-2184x^{2}y^{2}-1512x^{3}y^{4}-512x^{4}y^{3}-1224x^{4}y^{4}-500x+250ax+250bx+420xy^{2}-520xy^{3}+840axy^{2}+420bxy^{2}+520bxy^{3}+1092ax^{2}y^{2}+2016ax^{3}y^{4}-10206ax^{5}y^{4}+1638bx^{2}y^{2}+2016bx^{3}y^{4}+512bx^{4}y^{3}+1224bx^{4}y^{4}-10935bx^{5}y^{4}+17496abx^{5}y^{4}+720\right).$
  At x=1,y=1;
  $ND_{3}\left(\Gamma \right)=34992ab-8710b-12016a+3956$
5. $\left(D_{x}S_{y}+S_{x}D_{y}\right)\left(NM\left(\Gamma \right)\right)=\frac{x^{4}y^{5}}{1260}\left(25200x^{5}y^{4}-20400x^{2}y^{2}-7800x^{3}y^{4}-2520x^{4}y^{3}-5075x^{4}y^{4}-10080x+5040ax+5040bx+5328xy^{2}-5607xy^{3}+10656axy^{2}+5328bxy^{2}+5607bxy^{3}+10200ax^{2}y^{2}+10400ax^{3}y^{4}-35280ax^{5}y^{4}+15300bx^{2}y^{2}+10400bx^{3}y^{4}+2520bx^{4}y^{3}+5075bx^{4}y^{4}-37800bx^{5}y^{4}+60480abx^{5}y^{4}+20664\right)$
  At x=1,y=1;
  $ND_{5}\left(\Gamma \right)=\frac{254a}{315}+\frac{1147b}{126}+48ab-\frac{29}{126}$
6. $2S_{x}J\left(NM\left(\Gamma \right)\right)=2x^{10}\left(\frac{a}{5}+\frac{b}{5}-\frac{2}{5}\right)+2x^{12}\left(\frac{a}{3}+\frac{b}{6}+\frac{1}{6}\right)+2x^{16}\left(\frac{a}{4}+\frac{b}{4}-\frac{3}{16}\right)+2x^{13}\left(\frac{4a}{13}+\frac{6b}{13}-\frac{8}{13}\right)-2x^{18}\left(\frac{7a}{9}+\frac{5b}{6}-\frac{4ab}{3}-\frac{5}{9}\right)+2x^{13}\left(\frac{2b}{13}-\frac{2}{13}\right)+2x^{16}\left(\frac{b}{16}-\frac{1}{16}\right)+2x^{17}\left(\frac{2b}{17}-\frac{2}{17}\right)+\frac{16x^{9}}{9}$
  At x=1,y=1;
  $NH\left(\Gamma \right)=\frac{733a}{1170}+\frac{30703b}{26520}+\frac{8ab}{3}+\frac{2953}{19890}$
7. $S_{x}JD_{x}D_{y}\left(NM\left(\Gamma \right)\right)=\left(108ab-\frac{135b}{2}-63a+45\right)x^{18}+\left(\frac{144b}{17}-\frac{144}{17}\right)x^{17}+\left(\frac{63a}{4}+\frac{79b}{4}-\frac{253}{16}\right)x^{16}+\left(\frac{168a}{13}+\frac{332b}{13}-32\right)x^{13}+\left(\frac{35a}{3}+\frac{35b}{6}+\frac{35}{6}\right)x^{12}+\left(5a+5b-10\right)x^{10}+\frac{160x^{9}}{9}$
  At x=1,y=1;
  $NI\left(\Gamma \right)=108ab-\frac{7711b}{2652}-\frac{2755a}{156}+\frac{5699}{2448}$

□

**Theorem** **3.** *Let* Γ *be a graph of carbon nanosheet $HC_{5}C_{7}\left[a,b\right]$, then we have*

$NM\left(HC_{5}C_{7}\left[a,b\right]\right)=\left(24ab-14b-22a+13\right)x^{9}y^{9}+\left(8a+6b-12\right)x^{8}y^{9}+\left(4a+b\right)x^{8}y^{8}+\left(b+1\right)x^{7}y^{9}+\left(4a+2b-4\right)x^{6}y^{8}+2bx^{6}y^{7}+\left(4a+2b-2\right)x^{5}y^{8}+2x^{5}y^{7}+\left(2a-2\right)x^{5}y^{5}+\left(2b+4\right)x^{4}y^{5}+bx^{4}y^{4}.$

**Proof.**  By analyzing the construction of $HC_{5}C_{7}\left[a,b\right]$, the graph has 16ab+2a+4b vertices and 24ab+3b edges. The neighborhood degree of vertices of $HC_{5}C_{7}\left[a,b\right]$ can be 4,5,6,7,8 or 9 and hence the 11 partitions for the edge set based on the neighborhood degree are (4,4), (4,5), (5,5), (5,7), (5,8), (6,7), (6,8)(7,9), (8,8), (8,9) and (9,9). The edge partitions $\left(d_{x},d_{y}\right)$, $xy\in E(HC_{5}C_{7}\left[a,b\right])$ and the corresponding number of edges in each partition is given in Table 3.

From the definition of NM-polynomial of $HC_{5}C_{7}$ is obtained as,

$$\begin{array}{cc} NM(VC_{5}C_{7};x,y)= & \sum_{i\leq j}m_{\left(i,j\right)}x^{i}y^{j}\\ = & m_{\left(4,4\right)}x^{4}y^{4}+m_{\left(4,5\right)}x^{4}y^{5}+m_{\left(5,5\right)}x^{5}y^{5}+m_{\left(5,7\right)}x^{5}y^{7}+m_{\left(5,8\right)}x^{5}y^{8}+m_{\left(6,7\right)}x^{6}y^{7}+\\ & m_{\left(6,8\right)}x^{6}y^{8}+m_{\left(7,9\right)}x^{7}y^{9}+m_{\left(8,8\right)}x^{8}y^{8}+m_{\left(8,9\right)}x^{8}y^{9}+m_{\left(9,9\right)}x^{9}y^{9}\\ = & bx^{4}y^{4}+\left(2b+4\right)x^{4}y^{5}+\left(2a-2\right)x^{5}y^{5}+2x^{5}y^{7}+\left(2b+4a-2\right)x^{5}y^{8}+2bx^{6}y^{7}+\\ & \left(2b+4a-4\right)x^{6}y^{8}+\left(b+1\right)x^{7}y^{9}+\left(b+4a\right)x^{8}y^{8}+\left(6b+8a-12\right)x^{8}y^{9}+\\ & \left(24ab-14b-22a+13\right)x^{9}y^{9}\\ = & \left(24ab-14b-22a+13\right)x^{9}y^{9}+\left(8a+6b-12\right)x^{8}y^{9}+\\ & \left(4a+b\right)x^{8}y^{8}+\left(b+1\right)x^{7}y^{9}+\left(4a+2b-4\right)x^{6}y^{8}+2bx^{6}y^{7}+\\ & \left(4a+2b-2\right)x^{5}y^{8}+2x^{5}y^{7}+\left(2a-2\right)x^{5}y^{5}+\left(2b+4\right)x^{4}y^{5}+bx^{4}y^{4}. \end{array}$$

□

Now using this NM-polynomial, some neighborhood degree-based structural descriptors of $HC_{5}C_{7}$ is obtained.

**Theorem** **4.** *Let* Γ *be $HC_{5}C_{7}\left[a,b\right]$, then*

*1.*  $M_{1}^{\prime }\left(\Gamma \right)=432ab-12b-68a+4$
*2.*  $M_{2}^{nm}\left(\Gamma \right)=\frac{5357a}{32400}+\frac{44231b}{181440}+\frac{8ab}{27}+\frac{1517}{28350}$
*3.*  $NR_{\alpha }\left(\Gamma \right)=2^{6\alpha }\left(4a+b\right)+40^{\alpha }\left(4a+2b-2\right)+48^{\alpha }\left(4a+2b-4\right)+72^{\alpha }\left(8a+6b-12\right)+20^{\alpha }\left(2b+4\right)-3^{4\alpha }\left(22a+14b-24ab-13\right)+5^{2\alpha }\left(2a-2\right)+2\times 35^{\alpha }+2^{4\alpha }b+63^{\alpha }\left(b+1\right)+2^{\alpha +1}21^{\alpha }b$
*4.*  $ND_{3}\left(\Gamma \right)=34992ab-7072b-12920a+2606$
*5.*  $ND_{5}\left(\Gamma \right)=\frac{121a}{90}+\frac{311b}{45}+48ab-\frac{577}{1260}$
*6.*  $NH\left(\Gamma \right)=\frac{81247a}{139230}+\frac{27731b}{27846}+\frac{8ab}{3}+\frac{18709}{185640}$
*7.*  $NI\left(\Gamma \right)=108ab-\frac{831763b}{222768}-\frac{27994a}{1547}+\frac{327037}{222768}$

**Proof.**  Let $\Gamma =HC_{5}C_{7}$ Using the edge partition table given in Table 3, and using the expressions given in Table 1, various neighborhood degree based structural descriptors are obtained as follows;

We have;
$NM\left(\Gamma ;x,y\right)=\left(24ab-14b-22a+13\right)x^{9}y^{9}+\left(8a+6b-12\right)x^{8}y^{9}+\left(4a+b\right)x^{8}y^{8}+\left(b+1\right)x^{7}y^{9}+\left(4a+2b-4\right)x^{6}y^{8}+2bx^{6}y^{7}+\left(4a+2b-2\right)x^{5}y^{8}+2x^{5}y^{7}+\left(2a-2\right)x^{5}y^{5}+\left(2b+4\right)x^{4}y^{5}+bx^{4}y^{4}$
Then,

1. $\left(D_{x}+D_{y}\right)\left(NM\left(\Gamma \right)\right)=2x^{4}y^{4}\left(4b+18y-28x^{2}y^{4}+8x^{3}y^{5}-102x^{4}y^{5}+117x^{5}y^{5}+9by-10xy+12xy^{3}-13xy^{4}+26axy^{4}+13bxy^{4}+28ax^{2}y^{4}+32ax^{4}y^{4}+68ax^{4}y^{5}-198ax^{5}y^{5}+13bx^{2}y^{3}+14bx^{2}y^{4}+8bx^{3}y^{5}+8bx^{4}y^{4}+51bx^{4}y^{5}-126bx^{5}y^{5}+10axy+216abx^{5}y^{5}\right)$
  At x=1,y=1;
  $M_{1}^{\prime }\left(HC_{5}C_{7}\right)=432ab-12b-68a+4$
2. $\left(S_{x}S_{y}\right)\left(NM\left(\Gamma \right)\right)=\frac{\left(2x^{5}y^{7}\right)}{35}-\frac{\left(x^{9}y^{9}\left(22a+14b-24ab-13\right)\right)}{81}+\frac{\left(x^{5}y^{5}\left(2a-2\right)\right)}{25}+\frac{\left(x^{4}y^{5}\left(2b+4\right)\right)}{20}+\frac{\left(bx^{4}y^{4}\right)}{16}+\frac{\left(bx^{6}y^{7}\right)}{21}+\frac{\left(x^{8}y^{8}\left(4a+b\right)\right)}{64}+\frac{\left(x^{7}y^{9}\left(b+1\right)\right)}{63}+\frac{\left(x^{5}y^{8}\left(4a+2b-2\right)\right)}{40}+\frac{\left(x^{6}y^{8}\left(4a+2b-4\right)\right)}{48}+\frac{\left(x^{8}y^{9}\left(8a+6b-12\right)\right)}{72}$
  At x=1,y=1;
  $M_{2}^{nm}\left(HC_{5}C_{7}\right)=\frac{\left(5357a\right)}{32400}+\frac{\left(44231b\right)}{181440}+\frac{\left(8ab\right)}{27}+\frac{1517}{28350}$
3. $\left(D_{x}^{\alpha }+D_{y}^{\alpha }\right)\left(NM\left(\Gamma \right)\right)=-3^{4\alpha }\left(22a+14b-24ab-13\right)x^{9}y^{9}+72^{\alpha }\left(8a+6b-12\right)x^{8}y^{9}+2^{6\alpha }\left(4a+b\right)x^{8}y^{8}+63^{\alpha }\left(b+1\right)x^{7}y^{9}+48^{\alpha }\left(4a+2b-4\right)x^{6}y^{8}+2^{\alpha +1}21^{\alpha }bx^{6}y^{7}+40^{\alpha }\left(4a+2b-2\right)x^{5}y^{8}+2\times 35^{\alpha }x^{5}y^{7}+5^{2\alpha }\left(2a-2\right)x^{5}y^{5}+20^{\alpha }\left(2b+4\right)x^{4}y^{5}+2^{4\alpha }bx^{4}y^{4}$
  At x=1,y=1;
  $NR_{\alpha }\left(HC_{5}C_{7}\right)=2^{6\alpha }\left(4a+b\right)+40^{\alpha }\left(4a+2b-2\right)+48^{\alpha }\left(4a+2b-4\right)+72^{\alpha }\left(8a+6b-12\right)+20^{\alpha }\left(2b+4\right)-3^{\left(4\alpha \right)}\left(22a+14b-24ab-13\right)+5^{2\alpha }\left(2a-2\right)+2\times 35^{\alpha }+2^{4\alpha }b+63^{\alpha }\left(b+1\right)+2^{\alpha +1}21^{\alpha }b$
4. $D_{x}D_{y}\left(D_{x}+D_{y}\right)\left(NM\left(\Gamma \right)\right)=720x^{4}y^{5}-500x^{5}y^{5}+840x^{5}y^{7}-1040x^{5}y^{8}-2688x^{6}y^{8}+1008x^{7}y^{9}-14688x^{8}y^{9}+18954x^{9}y^{9}+500ax^{5}y^{5}+2080ax^{5}y^{8}+2688ax^{6}y^{8}+4096ax^{8}y^{8}+9792ax^{8}y^{9}-32076ax^{9}y^{9}+128bx^{4}y^{4}+360bx^{4}y^{5}+1040bx^{5}y^{8}+1092bx^{6}y^{7}+1344bx^{6}y^{8}+1008bx^{7}y^{9}+1024bx^{8}y^{8}+7344bx^{8}y^{9}-20412bx^{9}y^{9}+34992abx^{9}y^{9}$
  At x=1,y=1;
  $ND_{3}\left(HC_{5}C_{7}\right)=34992ab-7072b-12920a+2606$
5. $\left(D_{x}S_{y}+S_{x}D_{y}\right)\left(NM\left(\Gamma \right)\right)=\frac{x^{4}y^{4}}{1260}\left(2520b+10332y-10500x^{2}y^{4}+2600x^{3}y^{5}-30450x^{4}y^{5}+32760x^{5}y^{5}+5166by-5040xy+5328xy^{3}-5607xy^{4}+11214axy^{4}+5607bxy^{4}+10500ax^{2}y^{4}+10080ax^{4}y^{4}+20300ax^{4}y^{5}-55440ax^{5}y^{5}+5100bx^{2}y^{3}+5250bx^{2}y^{4}+2600bx^{3}y^{5}+2520bx^{4}y^{4}+15225bx^{4}y^{5}-35280bx^{5}y^{5}+5040axy+60480abx^{5}y^{5}\right)$
  At x=1,y=1;
  $ND_{5}\left(HC_{5}C_{7}\right)=\frac{121a}{90}+\frac{311b}{45}+48ab-\frac{577}{1260}$
6. $2S_{x}J\left(NM\left(\Gamma \right)\right)=2x^{16}\left(\frac{a}{4}+\frac{b}{16}\right)+2x^{14}\left(\frac{2a}{7}+\frac{b}{7}-\frac{2}{7}\right)+2x^{13}\left(\frac{4a}{13}+\frac{2b}{13}-\frac{2}{13}\right)+2x^{17}\left(\frac{8a}{17}+\frac{6b}{17}-\frac{12}{17}\right)-2x^{18}\left(\frac{11a}{9}+\frac{7b}{9}-\frac{4ab}{3}-\frac{13}{18}\right)+2x^{10}\left(\frac{a}{5}-\frac{1}{5}\right)+2x^{9}\left(\frac{2b}{9}+\frac{4}{9}\right)+2x^{16}\left(\frac{b}{16}+\frac{1}{16}\right)+\frac{bx^{8}}{4}+\frac{4bx^{13}}{13}+\frac{x^{12}}{3}$
  At x=1,y=1;
  $NH\left(HC_{5}C_{7}\right)=\frac{81247a}{139230}+\frac{27731b}{27846}+\frac{8ab}{3}+\frac{18709}{185640}$
7. $S_{x}JD_{x}D_{y}\left(NM\left(\Gamma \right)\right)=\left(108ab-63b-99a+\frac{117}{2}\right)x^{18}+\left(\frac{\left(576a\right)}{17}+\frac{\left(432b\right)}{17}-\frac{\left(864\right)}{17}\right)x^{17}+\left(16a+\frac{\left(127b\right)}{16}+\frac{63}{16}\right)x^{16}+\left(\frac{\left(96a\right)}{7}+\frac{\left(48b\right)}{7}-\frac{96}{7}\right)x^{14}+\left(\frac{\left(160a\right)}{13}+\frac{\left(164b\right)}{13}-\frac{80}{13}\right)x^{13}+\frac{\left(35x^{12}\right)}{6}+\left(5a-5\right)x^{10}+\left(\frac{\left(40b\right)}{9}+\frac{80}{9}\right)x^{9}+2bx^{8}$
  At x=1,y=1;
  $NI\left(HC_{5}C_{7}\right)=108ab-\frac{831763b}{222768}-\frac{27994a}{1547}+\frac{327037}{222768}$

□

## 4. Neighborhood Entropies of Pent-Heptagonal Nanosheets

Let NDT(Γ) denote the neighborhood degree based topological index of a graph Γ, then we get,

$$NDT\left(\Gamma \right)=\sum_{g\in E(\Gamma )}t\left(g\right)$$

where *t* is the functional characterizing the neighborhood degree-based topological index. The entropy measure [42,43,44] is denoted by $ENT_{NDT}\left(\Gamma \right)$ and is defined as,

(3) $$ENT_{NDT}\left(\Gamma \right)=log\left(NDT\left(\Gamma \right)\right)-\frac{1}{NDT(\Gamma )}log\left[\prod_{g\in E(\Gamma )}{\left[t\left(g\right)\right]}^{\left[t(g)\right]}\right].$$

### 4.1. ThirdVersion of the Zagreb Index $M_{1}^{\prime }$

From Theorems 2 and 3, the result is obtained as,

$$\begin{array}{cc} M_{1}^{\prime }\left(VC_{5}C_{7}\right)= & 432ab-8b-68a+8\;\mathrm{and}\\ M_{1}^{\prime }\left(HC_{5}C_{7}\right)= & 432ab-12b-68a+4. \end{array}$$

Since the number of edge partitions of $VC_{5}C_{7}$ and $HC_{5}C_{7}$ is 9 and 11, respectively, their corresponding entropy values are obtained.

Using the edge partition Table 2;

$$\begin{array}{cc} ENT_{M_{1}^{\prime }} & \left(VC_{5}C_{7}\right)=\log\left(M_{1}^{\prime }\left(VC_{5}C_{7}\right)\right)-\frac{1}{M_{1}^{\prime }\left(VC_{5}C_{7}\right)}\times \\ & \log\left[\begin{array}{c} \prod_{ij\in \chi_{\left\{4,5\right\}}}{\left[d_{i}+d_{j}\right]}^{\left[d_{i}+d_{j}\right]}\times \prod_{ij\in \chi_{\left\{5,5\right\}}}{\left[d_{i}+d_{j}\right]}^{\left[d_{i}+d_{j}\right]}\times \prod_{ij\in \chi_{\left\{5,7\right\}}}{\left[d_{i}+d_{j}\right]}^{\left[d_{i}+d_{j}\right]}\times \\ \prod_{ij\in \chi_{\left\{5,8\right\}}}{\left[d_{i}+d_{j}\right]}^{\left[d_{i}+d_{j}\right]}\times \prod_{ij\in \chi_{\left\{6,7\right\}}}{\left[d_{i}+d_{j}\right]}^{\left[d_{i}+d_{j}\right]}\times \prod_{ij\in \chi_{\left\{7,9\right\}}}{\left[d_{i}+d_{j}\right]}^{\left[d_{i}+d_{j}\right]}\times \\ \prod_{ij\in \chi_{\left\{8,8\right\}}}{\left[d_{i}+d_{j}\right]}^{\left[d_{i}+d_{j}\right]}\times \prod_{ij\in \chi_{\left\{8,9\right\}}}{\left[d_{i}+d_{j}\right]}^{\left[d_{i}+d_{j}\right]}\times \prod_{ij\in \chi_{\left\{9,9\right\}}}{\left[d_{i}+d_{j}\right]}^{\left[d_{i}+d_{j}\right]} \end{array}\right] \end{array}$$

$$\begin{array}{cc} ENT_{M_{1}^{\prime }} & \left(VC_{5}C_{7}\right)=\log\left(432ab-8b-68a+8\right)-\left(\frac{1}{432ab-8b-68a+8}\right)\times \\ & \log\left[\begin{array}{c} 9^{72}\times 10^{10(2b+2a-4)}\times 12^{12(2b+4a+2)}\times 13^{13(2b-2)}\times 13^{13(6b+4a-8)}\times \\ 16^{16(4b+4a-3)}\times 16^{16(b-1)}\times 17^{17(2b-2)}\times 18^{18(24ab-15b-14a+10)} \end{array}\right] \end{array}$$

Similarly using the edge partition Table 3,

$$\begin{array}{cc} ENT_{M_{1}^{\prime }} & \left(HC_{5}C_{7}\right)=\log\left(432ab-12b-68a+4\right)-\left(\frac{1}{432ab-12b-68a+4}\right)\times \\ & \log\left[\begin{array}{c} 8^{8b}\times 9^{9(2b+4)}\times 10^{10(2a-2)}\times 12^{24}\times 13^{13(2b+4a-2)}\times 13^{13(2b)}\times 14^{14(2b+4a-4)}\times \\ 16^{16(b+1)}\times 16^{16(b+4a)}\times 17^{17(6b+8a-12)}\times 18^{18(24ab-14b-22a+13)} \end{array}\right] \end{array}$$

### 4.2. Neighborhood Second Zagreb Index $M_{2}^{*}$

Here, we obtain the equations for calculating the entropy of Neighborhood Second Zagreb Index $M_{2}^{*}$ for the pent heptagonal nanosheets $VC_{5}C_{7}\left(a,b\right)$ and $HC_{5}C_{7}\left(a,b\right)$.

$$\begin{array}{cc} ENT_{M_{2}^{*}} & \left(VC_{5}C_{7}\right)=\log\left(M_{2}^{*}\left(VC_{5}C_{7}\right)\right)-\frac{1}{M_{2}^{*}\left(VC_{5}C_{7}\right)}\times \\ & \log\left[\begin{array}{c} \prod_{ij\in \chi_{\left\{4,5\right\}}}{\left[d_{i}d_{j}\right]}^{\left[d_{i}d_{j}\right]}\times \prod_{ij\in \chi_{\left\{5,5\right\}}}{\left[d_{i}d_{j}\right]}^{\left[d_{i}d_{j}\right]}\times \prod_{ij\in \chi_{\left\{5,7\right\}}}{\left[d_{i}d_{j}\right]}^{\left[d_{i}d_{j}\right]}\times \\ \prod_{ij\in \chi_{\left\{5,8\right\}}}{\left[d_{i}d_{j}\right]}^{\left[d_{i}d_{j}\right]}\times \prod_{ij\in \chi_{\left\{6,7\right\}}}{\left[d_{i}d_{j}\right]}^{\left[d_{i}d_{j}\right]}\times \prod_{ij\in \chi_{\left\{7,9\right\}}}{\left[d_{i}d_{j}\right]}^{\left[d_{i}d_{j}\right]}\times \\ \prod_{ij\in \chi_{\left\{8,8\right\}}}{\left[d_{i}d_{j}\right]}^{\left[d_{i}d_{j}\right]}\times \prod_{ij\in \chi_{\left\{8,9\right\}}}{\left[d_{i}d_{j}\right]}^{\left[d_{i}d_{j}\right]}\times \prod_{ij\in \chi_{\left\{9,9\right\}}}{\left[d_{i}d_{j}\right]}^{\left[d_{i}d_{j}\right]} \end{array}\right]\\ & =\log\left(1944ab-303b-524a+127\right)-\left(\frac{1}{1944ab-303b-524a+127}\right)\times \\ & \log\left[\begin{array}{c} 20^{160}\times 25^{25(2b+2a-4)}\times 35^{35(2b+4a+2)}\times 40^{40(2b-2)}\times 42^{42(6b+4a-8)}\times \\ 63^{63(4b+4a-3)}\times 64^{64(b-1)}\times 72^{72(2b-2)}\times 81^{81(24ab-15b-14a+10)} \end{array}\right] \end{array}$$

$$\begin{array}{cc} ENT_{M_{2}^{*}} & \left(HC_{5}C_{7}\right)=\log\left(1944ab-259b-548a+80\right)-\left(\frac{1}{1944ab-259b-548a+80}\right)\times \\ & \log\left[\begin{array}{c} 16^{16b}\times 20^{20(2b+4)}\times 25^{25(2a-2)}\times 35^{70}\times 40^{40(2b+4a-2)}\times 42^{42(2b)}\times 48^{48(2b+4a-4)}\times \\ 63^{63(b+1)}\times 64^{64(b+4a)}\times 72^{72(6b+8a-12)}\times 81^{81(24ab-14b-22a+13)} \end{array}\right] \end{array}$$

### 4.3. Neighborhood Forgotten Topological Index $F_{N}^{*}$

Here, we obtain the equations for calculating the entropy of Neighborhood Forgotten Topological Index $F_{N}^{*}$ for the pent heptagonal nanosheets $VC_{5}C_{7}\left(a,b\right)$ and $HC_{5}C_{7}\left(a,b\right)$.

$$\begin{array}{cc} ENT_{F_{N}^{*}} & \left(VC_{5}C_{7}\right)=\log\left(F_{N}^{*}\left(VC_{5}C_{7}\right)\right)-\frac{1}{F_{N}^{*}\left(VC_{5}C_{7}\right)}\times \\ & \log\left[\begin{array}{c} \prod_{ij\in \chi_{\left\{4,5\right\}}}{\left[d_{i}^{2}+d_{j}^{2}\right]}^{\left[d_{i}^{2}+d_{j}^{2}\right]}\times \prod_{ij\in \chi_{\left\{5,5\right\}}}{\left[d_{i}^{2}+d_{j}^{2}\right]}^{\left[d_{i}^{2}+d_{j}^{2}\right]}\times \prod_{ij\in \chi_{\left\{5,7\right\}}}{\left[d_{i}^{2}+d_{j}^{2}\right]}^{\left[d_{i}^{2}+d_{j}^{2}\right]}\times \\ \prod_{ij\in \chi_{\left\{5,8\right\}}}{\left[d_{i}^{2}+d_{j}^{2}\right]}^{\left[d_{i}^{2}+d_{j}^{2}\right]}\times \prod_{ij\in \chi_{\left\{6,7\right\}}}{\left[d_{i}^{2}+d_{j}^{2}\right]}^{\left[d_{i}^{2}+d_{j}^{2}\right]}\times \prod_{ij\in \chi_{\left\{7,9\right\}}}{\left[d_{i}^{2}+d_{j}^{2}\right]}^{\left[d_{i}^{2}+d_{j}^{2}\right]}\times \\ \prod_{ij\in \chi_{\left\{8,8\right\}}}{\left[d_{i}^{2}+d_{j}^{2}\right]}^{\left[d_{i}^{2}+d_{j}^{2}\right]}\times \prod_{ij\in \chi_{\left\{8,9\right\}}}{\left[d_{i}^{2}+d_{j}^{2}\right]}^{\left[d_{i}^{2}+d_{j}^{2}\right]}\times \prod_{ij\in \chi_{\left\{9,9\right\}}}{\left[d_{i}^{2}+d_{j}^{2}\right]}^{\left[d_{i}^{2}+d_{j}^{2}\right]} \end{array}\right]\\ = & \log\left(3888ab-556b-1012a+230\right)-\left(\frac{1}{3888ab-556b-1012a+230}\right)\times \\ & \log\left[\begin{array}{c} 41^{328}\times 50^{50(2b+2a-4)}\times 74^{74(2b+4a+2)}\times 89^{89(2b-2)}\times 85^{85(6b+4a-8)}\times \\ 130^{130(4b+4a-3)}\times 128^{128(b-1)}\times 145^{145(2b-2)}\times 162^{162(24ab-15b-14a+10)} \end{array}\right] \end{array}$$

$$\begin{array}{cc} ENT_{F_{N}^{*}} & \left(HC_{5}C_{7}\right)=\log\left(3888ab-478b-1036a+130\right)-\left(\frac{1}{3888ab-478b-1036a+130}\right)\times \\ & \log\left[\begin{array}{c} 32^{32b}\times 41^{41(2b+4)}\times 50^{50(2a-2)}\times 74^{148}\times 89^{89(2b+4a-2)}\times 85^{85(2b)}\times 100^{100(2b+4a-4)}\times \\ 130^{130(b+1)}\times 128^{128(b+4a)}\times 145^{145(6b+8a-12)}\times 162^{162(24ab-14b-22a+13)} \end{array}\right] \end{array}$$

### 4.4. Neighborhood Second Modified Zagreb Index $M_{2}^{nm}$

Here, we obtain the equations for calculating the entropy of Neighborhood Second Modified Zagreb Index $M_{2}^{nm}$ for the pent heptagonal nanosheets $VC_{5}C_{7}\left(a,b\right)$ and $HC_{5}C_{7}\left(a,b\right)$.

$$\begin{array}{cc} ENT_{M_{2}^{nm}} & \left(VC_{5}C_{7}\right)=\log\left(M_{2}^{nm}\left(VC_{5}C_{7}\right)\right)-\frac{1}{M_{2}^{nm}\left(VC_{5}C_{7}\right)}\times \\ & \log\left[\begin{array}{c} \prod_{ij\in \chi_{\left\{4,5\right\}}}{\left[\frac{1}{d_{i}d_{j}}\right]}^{\left[\frac{1}{d_{i}d_{j}}\right]}\times \prod_{ij\in \chi_{\left\{5,5\right\}}}{\left[\frac{1}{d_{i}d_{j}}\right]}^{\left[\frac{1}{d_{i}d_{j}}\right]}\times \prod_{ij\in \chi_{\left\{5,7\right\}}}{\left[\frac{1}{d_{i}d_{j}}\right]}^{\left[\frac{1}{d_{i}d_{j}}\right]}\times \\ \prod_{ij\in \chi_{\left\{5,8\right\}}}{\left[\frac{1}{d_{i}d_{j}}\right]}^{\left[\frac{1}{d_{i}d_{j}}\right]}\times \prod_{ij\in \chi_{\left\{6,7\right\}}}{\left[\frac{1}{d_{i}d_{j}}\right]}^{\left[\frac{1}{d_{i}d_{j}}\right]}\times \prod_{ij\in \chi_{\left\{7,9\right\}}}{\left[\frac{1}{d_{i}d_{j}}\right]}^{\left[\frac{1}{d_{i}d_{j}}\right]}\times \\ \prod_{ij\in \chi_{\left\{8,8\right\}}}{\left[\frac{1}{d_{i}d_{j}}\right]}^{\left[\frac{1}{d_{i}d_{j}}\right]}\times \prod_{ij\in \chi_{\left\{8,9\right\}}}{\left[\frac{1}{d_{i}d_{j}}\right]}^{\left[\frac{1}{d_{i}d_{j}}\right]}\times \prod_{ij\in \chi_{\left\{9,9\right\}}}{\left[\frac{1}{d_{i}d_{j}}\right]}^{\left[\frac{1}{d_{i}d_{j}}\right]} \end{array}\right]\\ & =\log\left(\frac{2554a}{14175}+\frac{76117b}{302400}+\frac{8ab}{27}+\frac{80833}{907200}\right)-\left(\frac{1}{\frac{2554a}{14175}+\frac{76117b}{302400}+\frac{8ab}{27}+\frac{80833}{907200}}\right)\times \\ & \;\log\left[\begin{array}{c} {\left(\frac{1}{20}\right)}^{\left(\frac{8}{20}\right)}\times {\left(\frac{1}{25}\right)}^{\frac{\left(2b+2a-4\right)}{25}}\times {\left(\frac{1}{35}\right)}^{\frac{\left(2b+4a+2\right)}{35}}\times {\left(\frac{1}{40}\right)}^{\frac{\left(2b-2\right)}{40}}\times {\left(\frac{1}{42}\right)}^{\frac{\left(6b+4a-8\right)}{42}}\times \\ {\left(\frac{1}{63}\right)}^{\frac{\left(4b+4a-3\right)}{63}}\times {\left(\frac{1}{64}\right)}^{\frac{\left(b-1\right)}{64}}\times {\left(\frac{1}{72}\right)}^{\frac{\left(2b-2\right)}{72}}\times {\left(\frac{1}{81}\right)}^{\frac{\left(24ab-15b-14a+10\right)}{81}} \end{array}\right] \end{array}$$

$$\begin{array}{cc} \; & ENT_{M_{2}^{nm}}\left(HC_{5}C_{7}\right)=\log\left(\frac{5357a}{32400}+\frac{44231b}{181440}+\frac{8ab}{27}+\frac{1517}{28350}\right)-\left(\frac{1}{\frac{5357a}{32400}+\frac{44231b}{181440}+\frac{8ab}{27}+\frac{1517}{28350}}\right)\times \\ & \log\left[\begin{array}{c} {\left(\frac{1}{16}\right)}^{\left(\frac{b}{16}\right)}\times {\left(\frac{1}{20}\right)}^{\frac{\left(2b+4\right)}{20}}\times {\left(\frac{1}{25}\right)}^{\frac{\left(2a-2\right)}{25}}\times {\left(\frac{1}{35}\right)}^{\left(\frac{2}{35}\right)}\times {\left(\frac{1}{40}\right)}^{\frac{\left(2b+4a-2\right)}{40}}\times {\left(\frac{1}{42}\right)}^{\frac{\left(2b\right)}{42}}\times \\ {\left(\frac{1}{48}\right)}^{\frac{\left(2b+4a-4\right)}{48}}\times {\left(\frac{1}{63}\right)}^{\frac{\left(b+1\right)}{63}}\times {\left(\frac{1}{64}\right)}^{\frac{\left(b+4a\right)}{64}}\times {\left(\frac{1}{72}\right)}^{\frac{\left(6b+8a-12\right)}{72}}\times {\left(\frac{1}{81}\right)}^{\frac{\left(24ab-14b-22a+13\right)}{81}} \end{array}\right] \end{array}$$

### 4.5. Neighborhood General Randic Index $NR_{\alpha }$

Here, we obtain the equations for calculating the entropy of Neighborhood General Randic Index $NR_{\alpha }$ for the pent heptagonal nanosheets $VC_{5}C_{7}\left(a,b\right)$ and $HC_{5}C_{7}\left(a,b\right)$.

$$\begin{array}{cc} ENT_{NR_{\alpha }} & \left(VC_{5}C_{7}\right)=\log\left(NR_{\alpha }\left(VC_{5}C_{7}\right)\right)-\frac{1}{NR_{\alpha }\left(VC_{5}C_{7}\right)}\times \\ & \log\left[\begin{array}{c} \prod_{ij\in \chi_{\left\{4,5\right\}}}{\left[d_{i}d_{j}\right]}^{\alpha {\left[d_{i}d_{j}\right]}^{\alpha }}\times \prod_{ij\in \chi_{\left\{5,5\right\}}}{\left[d_{i}d_{j}\right]}^{\alpha {\left[d_{i}d_{j}\right]}^{\alpha }}\times \prod_{ij\in \chi_{\left\{5,7\right\}}}{\left[d_{i}d_{j}\right]}^{\alpha {\left[d_{i}d_{j}\right]}^{\alpha }}\times \\ \prod_{ij\in \chi_{\left\{5,8\right\}}}{\left[d_{i}d_{j}\right]}^{\alpha {\left[d_{i}d_{j}\right]}^{\alpha }}\times \prod_{ij\in \chi_{\left\{6,7\right\}}}{\left[d_{i}d_{j}\right]}^{\alpha {\left[d_{i}d_{j}\right]}^{\alpha }}\times \prod_{ij\in \chi_{\left\{7,9\right\}}}{\left[d_{i}d_{j}\right]}^{\alpha {\left[d_{i}d_{j}\right]}^{\alpha }}\times \\ \prod_{ij\in \chi_{\left\{8,8\right\}}}{\left[d_{i}d_{j}\right]}^{\alpha {\left[d_{i}d_{j}\right]}^{\alpha }}\times \prod_{ij\in \chi_{\left\{8,9\right\}}}{\left[d_{i}d_{j}\right]}^{\alpha {\left[d_{i}d_{j}\right]}^{\alpha }}\times \prod_{ij\in \chi_{\left\{9,9\right\}}}{\left[d_{i}d_{j}\right]}^{\alpha {\left[d_{i}d_{j}\right]}^{\alpha }} \end{array}\right]\\ = & \log\left(NR_{\alpha }\left(VC_{5}C_{7}\right)\right)-\frac{1}{NR_{\alpha }\left(VC_{5}C_{7}\right)}\times \\ & \log\left[\begin{array}{c} {\left(20^{\alpha }\right)}^{8\times 20^{\alpha }}\times {\left(25^{\alpha }\right)}^{\left(25^{\alpha }\right)\left(2b+2a-4\right)}\times {\left(35^{\alpha }\right)}^{\left(35^{\alpha }\right)\left(2b+4a+2\right)}\times \\ {\left(40^{\alpha }\right)}^{\left(40^{\alpha }\right)\left(2b-2\right)}\times {\left(42^{\alpha }\right)}^{\left(42^{\alpha }\right)\left(6b+4a-8\right)}\times {\left(63^{\alpha }\right)}^{\left(63^{\alpha }\right)\left(4b+4a-3\right)}\times \\ {\left(64^{\alpha }\right)}^{\left(64^{\alpha }\right)\left(b-1\right)}\times {\left(72^{\alpha }\right)}^{\left(72^{\alpha }\right)\left(2b-2\right)}\times {\left(81^{\alpha }\right)}^{\left(81^{\alpha }\right)\left(24ab-15b-14a+10\right)} \end{array}\right] \end{array}$$

where $NR_{\alpha }\left(VC_{5}C_{7}\right)=35^{\alpha }\left(4a+2b+2\right)+42^{\alpha }\left(4a+6b-8\right)+63^{\alpha }\left(4a+4b-3\right)+2^{6\alpha }\left(b-1\right)+40^{\alpha }\left(2b-2\right)+72^{\alpha }\left(2b-2\right)+5^{2\alpha }\left(2a+2b-4\right)-3^{4\alpha }\left(14a+15b-24ab-10\right)+2^{2\alpha +3}5^{\alpha }.$

$$\begin{array}{cc} \; & ENT_{NR_{\alpha }}\left(HC_{5}C_{7}\right)=\log\left(NR_{\alpha }\left(HC_{5}C_{7}\right)\right)-\frac{1}{NR_{\alpha }\left(HC_{5}C_{7}\right)}\times \\ \; & \log\left[\begin{array}{c} {\left(16^{\alpha }\right)}^{(16^{\alpha })b}\times {\left(20^{\alpha }\right)}^{\left(20^{\alpha }\right)\left(2b+4\right)}\times {\left(25^{\alpha }\right)}^{\left(25^{\alpha }\right)\left(2a-2\right)}\times {\left(35^{\alpha }\right)}^{2\times (35^{\alpha })}\times \\ {\left(40^{\alpha }\right)}^{\left(40^{\alpha }\right)\left(2b+4a-2\right)}\times {\left(42^{\alpha }\right)}^{\left(42^{\alpha }\right)\left(2b\right)}\times {\left(48^{\alpha }\right)}^{\left(48^{\alpha }\right)\left(2b+4a-4\right)}\times {\left(63^{\alpha }\right)}^{\left(63^{\alpha }\right)\left(b+1\right)}\times \\ {\left(64^{\alpha }\right)}^{\left(64^{\alpha }\right)\left(b+4a\right)}\times {\left(72^{\alpha }\right)}^{\left(72^{\alpha }\right)\left(6b+8a-12\right)}\times {\left(81^{\alpha }\right)}^{\left(81^{\alpha }\right)\left(24ab-14b-22a+13\right)} \end{array}\right] \end{array}$$

where $NR_{\alpha }\left(HC_{5}C_{7}\right)=2^{6\alpha }\left(4a+b\right)+40^{\alpha }\left(4a+2b-2\right)+48^{\alpha }\left(4a+2b-4\right)+72^{\alpha }\left(8a+6b-12\right)+20^{\alpha }\left(2b+4\right)-3^{4\alpha }\left(22a+14b-24ab-13\right)+5^{2\alpha }\left(2a-2\right)+2\times 35^{\alpha }+2^{4\alpha }b+63^{\alpha }\left(b+1\right)+2^{\alpha +1}21^{\alpha }b.$

### 4.6. Third NDe Index $ND_{3}$

Here, we obtain the equations for calculating the entropy of Third NDe Index $ND_{3}$ for the pent heptagonal nanosheets $VC_{5}C_{7}\left(a,b\right)$ and $HC_{5}C_{7}\left(a,b\right)$.

$$\begin{array}{cc} ENT_{ND_{3}} & \left(VC_{5}C_{7}\right)=\log\left(ND_{3}\left(VC_{5}C_{7}\right)\right)-\frac{1}{ND_{3}\left(VC_{5}C_{7}\right)}\times \\ & \;\log\left[\begin{array}{c} \prod_{ij\in \chi_{\left\{4,5\right\}}}{\left[\left(d_{i}d_{j}\right)\left(d_{i}+d_{j}\right)\right]}^{\left(d_{i}d_{j}\right)\left(d_{i}+d_{j}\right)}\times \prod_{ij\in \chi_{\left\{5,5\right\}}}{\left[\left(d_{i}d_{j}\right)\left(d_{i}+d_{j}\right)\right]}^{\left(d_{i}d_{j}\right)\left(d_{i}+d_{j}\right)}\times \prod_{ij\in \chi_{\left\{5,7\right\}}}{\left[\left(d_{i}d_{j}\right)\left(d_{i}+d_{j}\right)\right]}^{\left(d_{i}d_{j}\right)\left(d_{i}+d_{j}\right)}\times \\ \prod_{ij\in \chi_{\left\{5,8\right\}}}{\left[\left(d_{i}d_{j}\right)\left(d_{i}+d_{j}\right)\right]}^{\left(d_{i}d_{j}\right)\left(d_{i}+d_{j}\right)}\times \prod_{ij\in \chi_{\left\{6,7\right\}}}{\left[\left(d_{i}d_{j}\right)\left(d_{i}+d_{j}\right)\right]}^{\left(d_{i}d_{j}\right)\left(d_{i}+d_{j}\right)}\times \prod_{ij\in \chi_{\left\{7,9\right\}}}{\left[\left(d_{i}d_{j}\right)\left(d_{i}+d_{j}\right)\right]}^{\left(d_{i}d_{j}\right)\left(d_{i}+d_{j}\right)}\times \\ \prod_{ij\in \chi_{\left\{8,8\right\}}}{\left[\left(d_{i}d_{j}\right)\left(d_{i}+d_{j}\right)\right]}^{(\left(d_{i}d_{j}\right)\left(d_{i}+d_{j}\right)}\times \prod_{ij\in \chi_{\left\{8,9\right\}}}{\left[\left(d_{i}d_{j}\right)\left(d_{i}+d_{j}\right)\right]}^{(\left(d_{i}d_{j}\right)\left(d_{i}+d_{j}\right)}\times \prod_{ij\in \chi_{\left\{9,9\right\}}}{\left[\left(d_{i}d_{j}\right)\left(d_{i}+d_{j}\right)\right]}^{\left(d_{i}d_{j}\right)\left(d_{i}+d_{j}\right)} \end{array}\right]\\ & \;=\log\left(34992ab-8710b-12016a+3956\right)-\left(\frac{1}{34992ab-8710b-12016a+3956}\right)\times \\ & \log\left[\begin{array}{c} 180^{1440}\times 250^{250(2b+2a-4)}\times 420^{420(2b+4a+2)}\times 520^{520(2b-2)}\times 546^{546(6b+4a-8)}\times \\ 1008^{1008(4b+4a-3)}\times 1024^{1024(b-1)}\times 1224^{1224(2b-2)}\times 1458^{1458(24ab-15b-14a+10)} \end{array}\right] \end{array}$$

$$\begin{array}{cc} ENT_{ND_{3}} & \left(HC_{5}C_{7}\right)=\log\left(34992ab-7072b-12920a+2606\right)-\left(\frac{1}{34992ab-7072b-12920a+2606}\right)\times \\ & \log\left[\begin{array}{c} 128^{128b}\times 180^{180(2b+4)}\times 250^{250(2a-2)}\times 420^{840}\times 520^{520(2b+4a-2)}\times 546^{546(2b)}\times \\ 672^{672(2b+4a-4)}\times 1008^{1008(b+1)}\times 1024^{1024(b+4a)}\times \\ 1224^{1224(6b+8a-12)}\times 1458^{1458(24ab-14b-22a+13)} \end{array}\right] \end{array}$$

### 4.7. Fifth NDe Index $ND_{5}$

Here, we obtain the equations for calculating the entropy of Fifth NDe Index $ND_{5}$ for the pent heptagonal nanosheets $VC_{5}C_{7}\left(a,b\right)$ and $HC_{5}C_{7}\left(a,b\right)$.

$$\begin{array}{cc} ENT_{ND_{5}} & \left(VC_{5}C_{7}\right)=\log\left(ND_{5}\left(VC_{5}C_{7}\right)\right)-\frac{1}{ND_{5}\left(VC_{5}C_{7}\right)}\times \\ & \;\log\left[\begin{array}{c} \prod_{ij\in \chi_{\left\{4,5\right\}}}{\left[\frac{d_{i}}{d_{j}}+\frac{d_{j}}{d_{i}}\right]}^{\left[\frac{d_{i}}{d_{j}}+\frac{d_{j}}{d_{i}}\right]}\times \prod_{ij\in \chi_{\left\{5,5\right\}}}{\left[\frac{d_{i}}{d_{j}}+\frac{d_{j}}{d_{i}}\right]}^{\left[\frac{d_{i}}{d_{j}}+\frac{d_{j}}{d_{i}}\right]}\times \prod_{ij\in \chi_{\left\{5,7\right\}}}{\left[\frac{d_{i}}{d_{j}}+\frac{d_{j}}{d_{i}}\right]}^{\left[\frac{d_{i}}{d_{j}}+\frac{d_{j}}{d_{i}}\right]}\times \\ \prod_{ij\in \chi_{\left\{5,8\right\}}}{\left[\frac{d_{i}}{d_{j}}+\frac{d_{j}}{d_{i}}\right]}^{\left[\frac{d_{i}}{d_{j}}+\frac{d_{j}}{d_{i}}\right]}\times \prod_{ij\in \chi_{\left\{6,7\right\}}}{\left[\frac{d_{i}}{d_{j}}+\frac{d_{j}}{d_{i}}\right]}^{\left[\frac{d_{i}}{d_{j}}+\frac{d_{j}}{d_{i}}\right]}\times \prod_{ij\in \chi_{\left\{7,9\right\}}}{\left[\frac{d_{i}}{d_{j}}+\frac{d_{j}}{d_{i}}\right]}^{\left[\frac{d_{i}}{d_{j}}+\frac{d_{j}}{d_{i}}\right]}\times \\ \prod_{ij\in \chi_{\left\{8,8\right\}}}{\left[\frac{d_{i}}{d_{j}}+\frac{d_{j}}{d_{i}}\right]}^{\left[\frac{d_{i}}{d_{j}}+\frac{d_{j}}{d_{i}}\right]}\times \prod_{ij\in \chi_{\left\{8,9\right\}}}{\left[\frac{d_{i}}{d_{j}}+\frac{d_{j}}{d_{i}}\right]}^{\left[\frac{d_{i}}{d_{j}}+\frac{d_{j}}{d_{i}}\right]}\times \prod_{ij\in \chi_{\left\{9,9\right\}}}{\left[\frac{d_{i}}{d_{j}}+\frac{d_{j}}{d_{i}}\right]}^{\left[\frac{d_{i}}{d_{j}}+\frac{d_{j}}{d_{i}}\right]} \end{array}\right]\\ & =\log\left(\frac{254a}{315}+\frac{1147b}{126}+48ab-\frac{29}{126}\right)-\left(\frac{1}{\frac{254a}{315}+\frac{1147b}{126}+48ab-\frac{29}{126}}\right)\times \\ & \log\left[\begin{array}{c} {\left(\frac{41}{20}\right)}^{\left(\frac{328}{20}\right)}\times 2^{2(2b+2a-4)}\times {\left(\frac{74}{35}\right)}^{\frac{74(2b+4a+2)}{35}}\times {\left(\frac{89}{40}\right)}^{\frac{89(2b-2)}{40}}\times {\left(\frac{85}{42}\right)}^{\frac{85(6b+4a-8)}{42}}\times \\ {\left(\frac{130}{63}\right)}^{\frac{130(4b+4a-3)}{63}}\times 2^{\left(b-1\right)}\times {\left(\frac{145}{72}\right)}^{\frac{145(2b-2)}{72}}\times 2^{2(24ab-15b-14a+10)} \end{array}\right] \end{array}$$

$$\begin{array}{cc} ENT_{ND_{5}} & \left(HC_{5}C_{7}\right)=\log\left(\frac{121a}{90}+\frac{311b}{45}+48ab-\frac{577}{1260}\right)-\left(\frac{1}{\frac{121a}{90}+\frac{311b}{45}+48ab-\frac{577}{1260}}\right)\times \\ & \;\log\left[\begin{array}{c} 2^{2b}\times {\left(\frac{41}{20}\right)}^{\frac{41(2b+4)}{20}}\times 2^{2(2a-2)}\times {\left(\frac{74}{35}\right)}^{\frac{148}{35}}\times {\left(\frac{89}{40}\right)}^{\frac{89(2b+4a-2)}{40}}\times {\left(\frac{85}{42}\right)}^{\frac{85(2b)}{42}}\times \\ {\left(\frac{100}{48}\right)}^{\frac{100(2b+4a-4)}{48}}\times {\left(\frac{130}{63}\right)}^{\frac{130(b+1)}{63}}\times 2^{2(b+4a)}\times {\left(\frac{145}{72}\right)}^{\frac{145(6b+8a-12)}{72}}\times 2^{2(24ab-14b-22a+13)} \end{array}\right] \end{array}$$

### 4.8. Neighborhood Harmonic Index NH

Here, we obtain the equations for calculating the entropy of Neighborhood Harmonic Index NH for the pent heptagonal nanosheets $VC_{5}C_{7}\left(a,b\right)$ and $HC_{5}C_{7}\left(a,b\right)$.

$$\begin{array}{cc} ENT_{NH} & \left(VC_{5}C_{7}\right)=\log\left(NH\left(VC_{5}C_{7}\right)\right)-\frac{1}{NH(VC_{5}C_{7})}\times \\ & \log\left[\begin{array}{c} \prod_{ij\in \chi_{\left\{4,5\right\}}}{\left[\frac{2}{d_{i}+d_{j}}\right]}^{\left[\frac{2}{d_{i}+d_{j}}\right]}\times \prod_{ij\in \chi_{\left\{5,5\right\}}}{\left[\frac{2}{d_{i}+d_{j}}\right]}^{\left[\frac{2}{d_{i}+d_{j}}\right]}\times \prod_{ij\in \chi_{\left\{5,7\right\}}}{\left[\frac{2}{d_{i}+d_{j}}\right]}^{\left[\frac{2}{d_{i}+d_{j}}\right]}\times \\ \prod_{ij\in \chi_{\left\{5,8\right\}}}{\left[\frac{2}{d_{i}+d_{j}}\right]}^{\left[\frac{2}{d_{i}+d_{j}}\right]}\times \prod_{ij\in \chi_{\left\{6,7\right\}}}{\left[\frac{2}{d_{i}+d_{j}}\right]}^{\left[\frac{2}{d_{i}+d_{j}}\right]}\times \prod_{ij\in \chi_{\left\{7,9\right\}}}{\left[\frac{2}{d_{i}+d_{j}}\right]}^{\left[\frac{2}{d_{i}+d_{j}}\right]}\times \\ \prod_{ij\in \chi_{\left\{8,8\right\}}}{\left[\frac{2}{d_{i}+d_{j}}\right]}^{\left[\frac{2}{d_{i}+d_{j}}\right]}\times \prod_{ij\in \chi_{\left\{8,9\right\}}}{\left[\frac{2}{d_{i}+d_{j}}\right]}^{\left[\frac{2}{d_{i}+d_{j}}\right]}\times \prod_{ij\in \chi_{\left\{9,9\right\}}}{\left[\frac{2}{d_{i}+d_{j}}\right]}^{\left[\frac{2}{d_{i}+d_{j}}\right]} \end{array}\right]\\ = & \log\left(\frac{733a}{1170}+\frac{30703b}{26520}+\frac{8ab}{3}+\frac{2953}{19890}\right)-\left(\frac{1}{\frac{733a}{1170}+\frac{30703b}{26520}+\frac{8ab}{3}+\frac{2953}{19890}}\right)\times \\ & \log\left[\begin{array}{c} {\left(\frac{2}{9}\right)}^{\frac{16}{9}}\times {\left(\frac{1}{5}\right)}^{\frac{\left(2b+2a-4\right)}{5}}\times {\left(\frac{1}{6}\right)}^{\frac{\left(2b+4a+2\right)}{6}}\times {\left(\frac{2}{13}\right)}^{\frac{2(2b-2)}{13}}\times {\left(\frac{2}{13}\right)}^{\frac{2(6b+4a-8)}{13}}\times \\ {\left(\frac{1}{8}\right)}^{\frac{1(4b+4a-3)}{8}}\times {\left(\frac{1}{8}\right)}^{\frac{\left(b-1\right)}{8}}\times {\left(\frac{1}{17}\right)}^{\frac{\left(2b-2\right)}{17}}\times {\left(\frac{1}{9}\right)}^{\frac{\left(24ab-15b-14a+10\right)}{9}} \end{array}\right] \end{array}$$

$$\begin{array}{cc} \;ENT_{NH} & \left(HC_{5}C_{7}\right)=\log\left(\frac{81247a}{139230}+\frac{27731b}{27846}+\frac{8ab}{3}+\frac{18709}{185640}\right)-\left(\frac{1}{\frac{81247a}{139230}+\frac{27731b}{27846}+\frac{8ab}{3}+\frac{18709}{185640}}\right)\times \\ & \log\left[\begin{array}{c} {\left(\frac{1}{4}\right)}^{\frac{b}{4}}\times {\left(\frac{2}{9}\right)}^{\frac{2(2b+4)}{9}}\times {\left(\frac{1}{5}\right)}^{\frac{\left(2a-2\right)}{5}}\times {\left(\frac{1}{6}\right)}^{\left(\frac{1}{3}\right)}\times {\left(\frac{2}{13}\right)}^{\frac{2(2b+4a-2)}{13}}\times {\left(\frac{2}{13}\right)}^{\frac{2(2b)}{13}}\times \\ {\left(\frac{1}{7}\right)}^{\frac{\left(2b+4a-4\right)}{7}}\times {\left(\frac{1}{8}\right)}^{\frac{\left(b+1\right)}{8}}\times {\left(\frac{1}{8}\right)}^{\frac{\left(b+4a\right)}{8}}\times {\left(\frac{2}{17}\right)}^{\frac{2(6b+8a-12)}{17}}\times {\left(\frac{1}{9}\right)}^{\frac{\left(24ab-14b-22a+13\right)}{9}} \end{array}\right] \end{array}$$

### 4.9. Neighborhood Inverse Sum Index NI

Here, we obtain the equations for calculating the entropy of Neighborhood Inverse Sum Index NI for the pent heptagonal nanosheets $VC_{5}C_{7}\left(a,b\right)$ and $HC_{5}C_{7}\left(a,b\right)$.

$$\begin{array}{cc} ENT_{NI} & \left(VC_{5}C_{7}\right)=\log\left(NI\left(VC_{5}C_{7}\right)\right)-\frac{1}{NI(VC_{5}C_{7})}\times \\ & \log\left[\begin{array}{c} \prod_{ij\in \chi_{\left\{4,5\right\}}}{\left[\frac{d_{i}d_{j}}{d_{i}+d_{j}}\right]}^{\left(\frac{d_{i}d_{j}}{d_{i}+d_{j}}\right)}\times \prod_{ij\in \chi_{\left\{5,5\right\}}}{\left[\frac{d_{i}d_{j}}{d_{i}+d_{j}}\right]}^{\left(\frac{d_{i}d_{j}}{d_{i}+d_{j}}\right)}\times \prod_{ij\in \chi_{\left\{5,7\right\}}}{\left[\frac{d_{i}d_{j}}{d_{i}+d_{j}}\right]}^{\left(\frac{d_{i}d_{j}}{d_{i}+d_{j}}\right)}\times \\ \prod_{ij\in \chi_{\left\{5,8\right\}}}{\left[\frac{d_{i}d_{j}}{d_{i}+d_{j}}\right]}^{\left(\frac{d_{i}d_{j}}{d_{i}+d_{j}}\right)}\times \prod_{ij\in \chi_{\left\{6,7\right\}}}{\left[\frac{d_{i}d_{j}}{d_{i}+d_{j}}\right]}^{\left(\frac{d_{i}d_{j}}{d_{i}+d_{j}}\right)}\times \prod_{ij\in \chi_{\left\{7,9\right\}}}{\left[\frac{d_{i}d_{j}}{d_{i}+d_{j}}\right]}^{\left(\frac{d_{i}d_{j}}{d_{i}+d_{j}}\right)}\times \\ \prod_{ij\in \chi_{\left\{8,8\right\}}}{\left[\frac{d_{i}d_{j}}{d_{i}+d_{j}}\right]}^{\left(\frac{d_{i}d_{j}}{d_{i}+d_{j}}\right)}\times \prod_{ij\in \chi_{\left\{8,9\right\}}}{\left[\frac{d_{i}d_{j}}{d_{i}+d_{j}}\right]}^{\left(\frac{d_{i}d_{j}}{d_{i}+d_{j}}\right)}\times \prod_{ij\in \chi_{\left\{9,9\right\}}}{\left[\frac{d_{i}d_{j}}{d_{i}+d_{j}}\right]}^{\left(\frac{d_{i}d_{j}}{d_{i}+d_{j}}\right)} \end{array}\right]\\ = & \log\left(108ab-\frac{7711b}{2652}-\frac{2755a}{156}+\frac{5699}{2448}\right)-\left(\frac{1}{108ab-\frac{7711b}{2652}-\frac{2755a}{156}+\frac{5699}{2448}}\right)\times \\ & \;\log\left[\begin{array}{c} {\left(\frac{20}{9}\right)}^{\frac{160}{9}}\times {\left(\frac{5}{2}\right)}^{\frac{5(2b+2a-4)}{2}}\times {\left(\frac{35}{12}\right)}^{\frac{35(2b+4a+2)}{12}}\times {\left(\frac{40}{13}\right)}^{\frac{40(2b-2)}{13}}\times {\left(\frac{42}{13}\right)}^{\frac{42(6b+4a-8)}{13}}\times \\ {\left(\frac{63}{16}\right)}^{\frac{63(4b+4a-3)}{16}}\times 4^{4(b-1)}\times {\left(\frac{72}{17}\right)}^{\frac{72(2b-2)}{17}}\times {\left(\frac{9}{2}\right)}^{\frac{9(24ab-15b-14a+10)}{2}} \end{array}\right] \end{array}$$

$$\begin{array}{cc} \;ENT_{NI} & \left(HC_{5}C_{7}\right)=\log\left(108ab-\frac{831763b}{222768}-\frac{27994a}{1547}+\frac{327037}{222768}\right)-\left(\frac{1}{108ab-\frac{831763b}{222768}-\frac{27994a}{1547}+\frac{327037}{222768}}\right)\times \\ & \log\left[\begin{array}{c} 2^{2b}\times {\left(\frac{20}{9}\right)}^{\frac{20(2b+4)}{9}}\times {\left(\frac{5}{2}\right)}^{\frac{5(2a-2)}{2}}\times {\left(\frac{35}{12}\right)}^{\left(\frac{35}{6}\right)}\times {\left(\frac{40}{13}\right)}^{\frac{40(2b+4a-2)}{13}}\times {\left(\frac{42}{13}\right)}^{\frac{42(2b)}{13}}\times \\ {\left(\frac{24}{7}\right)}^{\frac{24(2b+4a-4)}{7}}\times {\left(\frac{63}{16}\right)}^{\frac{63(b+1)}{16}}\times 4^{4(b+4a)}\times {\left(\frac{72}{17}\right)}^{\frac{72(6b+8a-12)}{17}}\times {\left(\frac{9}{2}\right)}^{\frac{9(24ab-14b-22a+13)}{2}} \end{array}\right] \end{array}$$

### 4.10. Sanskruthi Index *S*

Here, we obtain the equations for calculating the entropy of Sanskruthi Index *S* for the pent heptagonal nanosheets $VC_{5}C_{7}\left(a,b\right)$ and $HC_{5}C_{7}\left(a,b\right)$.

$$\begin{array}{cc} \; & \;ENT_{S}\left(VC_{5}C_{7}\right)=\log\left(S\left(VC_{5}C_{7}\right)\right)-\frac{1}{S(VC_{5}C_{7})}\times \\ & \;\log\left[\begin{array}{c} \prod_{ij\in \chi_{\left\{4,5\right\}}}{\left[{\left(\frac{d_{i}d_{j}}{d_{i}+d_{j}-2}\right)}^{3}\right]}^{{\left[\frac{d_{i}d_{j}}{d_{i}+d_{j}-2}\right]}^{3}}\times \prod_{ij\in \chi_{\left\{5,5\right\}}}{\left[{\left(\frac{d_{i}d_{j}}{d_{i}+d_{j}-2}\right)}^{3}\right]}^{{\left[\frac{d_{i}d_{j}}{d_{i}+d_{j}-2}\right]}^{3}}\times \prod_{ij\in \chi_{\left\{5,7\right\}}}{\left[{\left(\frac{d_{i}d_{j}}{d_{i}+d_{j}-2}\right)}^{3}\right]}^{{\left[\frac{d_{i}d_{j}}{d_{i}+d_{j}-2}\right]}^{3}}\times \\ \prod_{ij\in \chi_{\left\{5,8\right\}}}{\left[{\left(\frac{d_{i}d_{j}}{d_{i}+d_{j}-2}\right)}^{3}\right]}^{{\left[\frac{d_{i}d_{j}}{d_{i}+d_{j}-2}\right]}^{3}}\times \prod_{ij\in \chi_{\left\{6,7\right\}}}{\left[{\left(\frac{d_{i}d_{j}}{d_{i}+d_{j}-2}\right)}^{3}\right]}^{{\left[\frac{d_{i}d_{j}}{d_{i}+d_{j}-2}\right]}^{3}}\times \prod_{ij\in \chi_{\left\{7,9\right\}}}{\left[{\left(\frac{d_{i}d_{j}}{d_{i}+d_{j}-2}\right)}^{3}\right]}^{{\left[\frac{d_{i}d_{j}}{d_{i}+d_{j}-2}\right]}^{3}}\times \\ \prod_{ij\in \chi_{\left\{8,8\right\}}}{\left[{\left(\frac{d_{i}d_{j}}{d_{i}+d_{j}-2}\right)}^{3}\right]}^{{\left[\frac{d_{i}d_{j}}{d_{i}+d_{j}-2}\right]}^{3}}\times \prod_{ij\in \chi_{\left\{8,9\right\}}}{\left[{\left(\frac{d_{i}d_{j}}{d_{i}+d_{j}-2}\right)}^{3}\right]}^{{\left[\frac{d_{i}d_{j}}{d_{i}+d_{j}-2}\right]}^{3}}\times \prod_{ij\in \chi_{\left\{9,9\right\}}}{\left[{\left(\frac{d_{i}d_{j}}{d_{i}+d_{j}-2}\right)}^{3}\right]}^{{\left[\frac{d_{i}d_{j}}{d_{i}+d_{j}-2}\right]}^{3}} \end{array}\right]\\ & \;=\log\left(S\left(VC_{5}C_{7}\right)\right)-\frac{1}{S(VC_{5}C_{7})}\times \\ & \;\log\left[\begin{array}{c} {\left(\frac{8000}{343}\right)}^{\frac{64000}{343}}\times {\left(\frac{15625}{512}\right)}^{\frac{15625(2b+2a-4)}{512}}\times {\left(\frac{343}{8}\right)}^{\frac{343(2b+4a+2)}{8}}\times {\left(\frac{64000}{1331}\right)}^{\frac{64000(2b-2)}{1331}}\times {\left(\frac{74088}{1331}\right)}^{\frac{74088(6b+4a-8)}{1331}}\times \\ {\left(\frac{729}{8}\right)}^{\frac{729(4b+4a-3)}{8}}\times {\left(\frac{32768}{343}\right)}^{\frac{32768(b-1)}{343}}\times {\left(\frac{13824}{125}\right)}^{\frac{13824(2b-2)}{125}}\times {\left(\frac{531441}{4096}\right)}^{\frac{531441(24ab-15b-14a+10)}{4096}} \end{array}\right] \end{array}$$

where $S\left(VC_{5}C_{7}\right)=\frac{1594323ab}{512}-\frac{14620607759501b}{21249536000}-\frac{2717055933a}{2725888}+\frac{36950846358693}{116872448000}$.

$$\begin{array}{cc} \; & ENT_{S}\left(HC_{5}C_{7}\right)=\log\left(S\left(HC_{5}C_{7}\right)\right)-\frac{1}{S(HC_{5}C_{7})}\times \\ \log & \left[\begin{array}{c} {\left(\frac{512}{27}\right)}^{\frac{512b}{27}}\times {\left(\frac{8000}{343}\right)}^{\frac{8000(2b+4)}{343}}\times {\left(\frac{15625}{512}\right)}^{\frac{15625(2a-2)}{512}}\times {\left(\frac{343}{8}\right)}^{\left(\frac{343}{4}\right)}\times {\left(\frac{64000}{1331}\right)}^{\frac{64000(2b+4a-2)}{1331}}\times {\left(\frac{74088}{1331}\right)}^{\frac{74088(2b)}{1331}}\\ \times 64^{64(2b+4a-4)}\times {\left(\frac{729}{8}\right)}^{\frac{729(b+1)}{8}}\times {\left(\frac{32768}{343}\right)}^{\frac{32768(b+4a)}{343}}\times {\left(\frac{13824}{125}\right)}^{\frac{13824(6b+8a-12)}{125}}\times {\left(\frac{531441}{4096}\right)}^{\frac{531441(24ab-14b-22a+13)}{4096}} \end{array}\right] \end{array}$$

where $S\left(HC_{5}C_{7}\right)=\frac{1594323ab}{512}-\frac{1783308545665133b}{3155556096000}-\frac{126009252864147a}{116872448000}+\frac{50620823728941}{233744896000}$.

## 5. Applications of Molecular Descriptors and M-Polynomial Method

**Figure 5:**
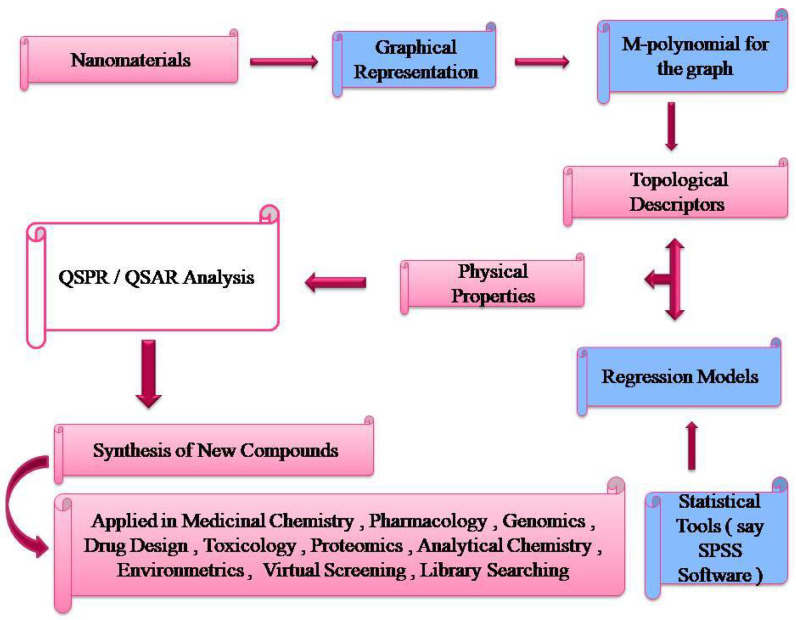
Flowchart relating Topological Descriptors and its potential uses.

Molecular Descriptors have been found to exhibit strong correlations with such a wide range of biological and physico-chemical characteristics, indicating that they are information-rich, typically rapid and simple to evaluate. As a result, they serve as effective descriptors in QSARs and QSPRs which are used for predicting the toxicity of a chemical or the potency of a medicine for upcoming release on the market. One of the most significant descriptors for medication design is physicochemical attributes. The foundation of QSARs is the idea that a molecule’s structure must contain elements responsible for its physical, chemical and biological characteristics and the efficiency to encode these characteristics in single or several descriptors. For usage in QSAR/QSPR modeling, thousands of physicochemical as well as structural descriptors are already accessible. The great majority of these values are computed because experimentation requires intense labor and expensive equipmentation, whereas computation can be done quickly and cheaply using a variety of software that is currently accessible. Figure 5 is a flow chart illustrating the relationship between topological descriptors and potential uses.

The expression given below is used to correlate different physical characteristics of different molecules using these descriptors. Following is the linear regression model,

(4) Q=d+c(TI)

where *Q* is a physical property of the compound, *c* is the regression coefficient, *d* is a constant, and *TI* is a topological descriptor. The regression coefficient *c* and invariant D are computed using software (say SPSS software) [45,46,47]. Theoretical analysis might assist those working in the pharmaceutical sector, including chemists, in predicting a compound’s features without experimentation. Additionally, it aids in the development of novel compounds with the required characteristics. The M- polynomial technique makes it simpler and shorter to calculate descriptors from the carbon sheet than the conventional algorithmic method does. This is due to the fact that the M- polynomial is distinct and may be used to compute numerous descriptors by using differentiation, integration or a combination of both instead of calculating each one separately. Further information on the dependence of physico-chemical characteristics on topological components, the different aspects of the electro-topological state index for atoms in molecules and the characterization of molecular branching can be seen in [33,34,36,37].

### 5.1. Prediction of Properties Using Neighborhood Degree Descriptors

**Figure 6:**
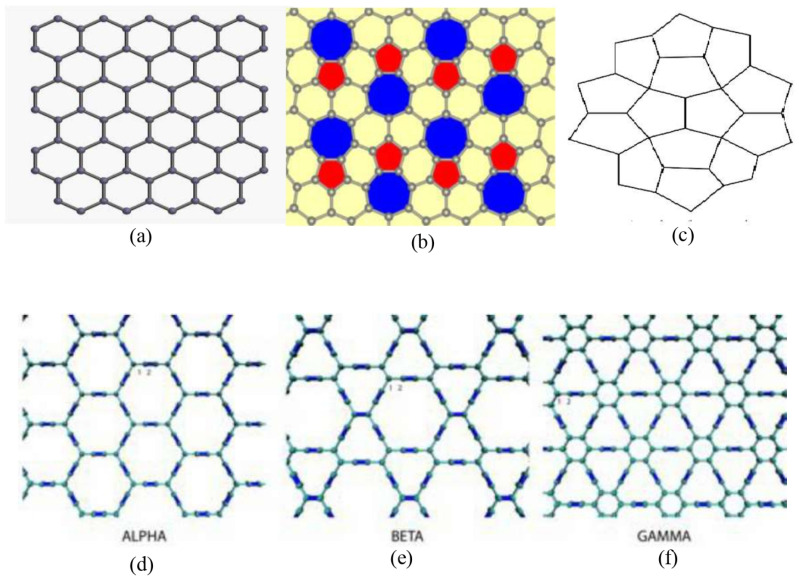
Graphene and its derivatives; (**a**) Graphene (**b**) Phagraphene (**c**) Penta Graphene (**d**) α graphyne (**e**) β graphyne (**f**) γ graphyne.

Since Graphene was discovered in 2004, it has sparked a great deal of curiosity among people all around the world. Many investigations have been done to investigate its features and prospective uses. In addition to the graphene boom, significant efforts have been undertaken to identify its allotropes as well. Pent heptagonal carbon nanosheet, also known as Pentaheptite graphene, is an allotrope of Graphene [48]. This section explores the predictive potential of some properties of the graphene derivatives using neighborhood degree topological descriptors in conjunction with the data on some graphene allotropes. Further information relating to the properties of graphene and its allotropes can be obtained from [49,50]. Figure 6 shows graphene and some of its derivatives.

We investigate Young’s modulus (E) and Poisson’s ratio (ν) for the graphene structures. Young’s modulus is a measurement of a material’s capacity to endure changes in length when subjected to lengthwise tension or compression and Poisson’s ratio is the ratio of a material’s transverse contraction to its longitudinal extension strain when subjected to stretching forces. Table 4 summarises the data available for Young’s modulus and Poisson ratio of graphene derivatives [51,52,53].

### 5.2. Linear Regression Models for Various Descriptors

We employ the Least Squares Approximation approach to determine the relationship between neighborhood degree topological descriptors and characteristics of chemical compounds. To derive the equation connecting descriptors and properties, we calculate the correlation coefficient and perform regression analysis. Correlation is a statistical term that describes how closely two variables are linearly connected. The correlation coefficient has a value between −1 and 1. 1 indicates a perfect positive correlation, −1 indicates a perfect negative correlation, and 0 indicates no correlation. We will perform the regression analysis for the derivatives of graphene excluding Pentaheptite graphene and then construct the linear expression relating descriptors and properties. Further, we compare the experimentally observed values with the ones predicted for the pent heptagonal nanosheet [32].

The neighborhood degree descriptors for the core structures of graphene and its allotropes are tabulated in Table 5. By fitting using the Least Square Approximation technique, the correlation coefficients obtained for graphene allotropes are tabulated in Table 6. Here, topological descriptors and properties serve as the horizontal and vertical data sets, respectively.

In accordance with the correlation table, these allotropes show a strong correlation within themselves for both attributes. The neighborhood second modified Zagreb index shows a strong correlation compared to other indices for Young’s modulus and the third version of the Zagreb index appears to have a significantly better correlation for Poisson’s ratio. Using the data analysis tools in Microsoft Excel and modeling in accordance with Equation (4), we arrive at the following linear regression model for the properties using descriptors.

For Young’s modulus, we obtain the model as

$$E=352.714+\left(-85.583\right)\times M_{2}^{nm}$$

where *E* is Young’s modulus and $M_{2}^{nm}$ is the neighborhood second modified Zagreb index. Similarly for the Poisson’s ratio, the linear Regression model is obtained as

$$\nu =\left(-0.3866\right)+\left(1.4833\times 10^{-3}\right)\times M_{1}^{\prime }$$

where ν is the Poisson’s ratio and $M_{1}^{\prime }$ is the third version of the Zagreb index.

**Figure 7:**
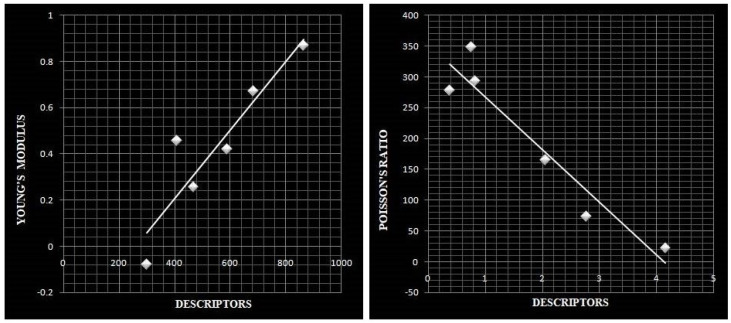
Scatter Diagram for the properties.

This model gives values 283 N/m and 0.15 for Young’s modulus (*E*) and Poisson’s Ratio (ν), respectively, for pentaheptite graphene. The experimental value of Pentaheptite graphene, which is shown in Table 4, closely follows the expected values. Moreover, we can also predict Young’s modulus and Poisson’s ratio values of various other derivatives of graphene successfully using this linear regression model. We can even forecast the characteristics of molecules with greater dimensions with the appropriate regression model. The Scatter plots for the highest correlated regression models can be seen in Figure 7.

## 6. Comparison of Various Indices and Their Entropy Measures

**Figure 8:**
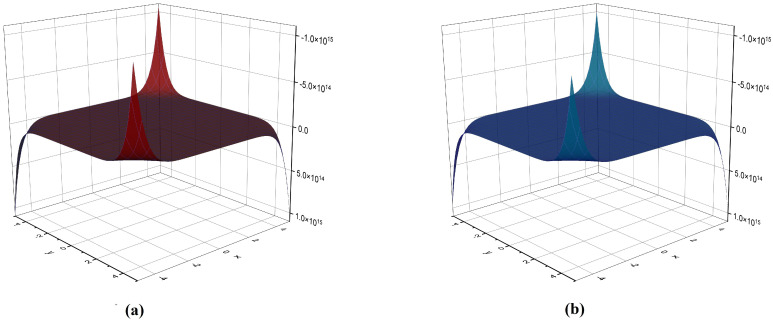
The *NM* polynomial graph of (**a**) $VC_{5}C_{7}\left(4,4\right)$ and (**b**) $HC_{5}C_{7}\left(4,4\right)$.

**Figure 9:**
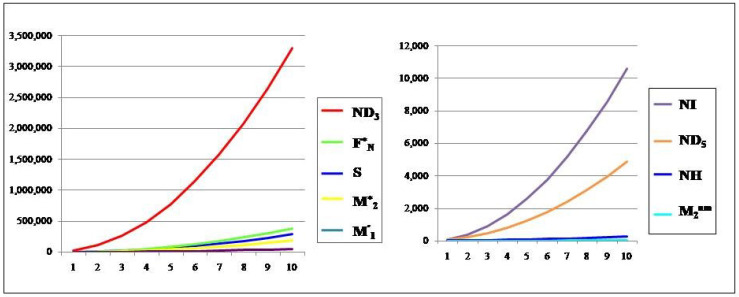
The neighborhood degree indices of $VC_{5}C_{7}\left(a,a\right)$.

**Figure 10:**
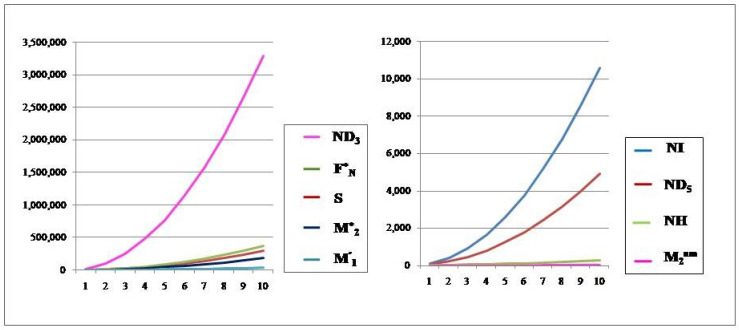
The neighborhood degree indices of $HC_{5}C_{7}\left(a,a\right)$.

**Figure 11:**
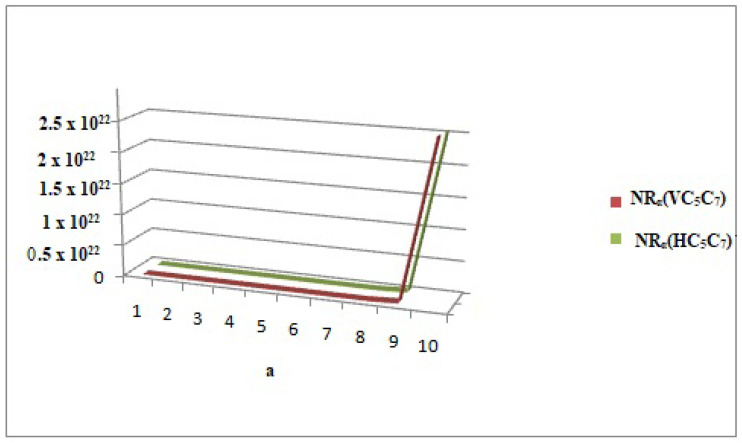
Comparison graph of neighborhood general Randic index of $VC_{5}C_{7}\left(a,a\right)$ and $HC_{5}C_{7}\left(a,a\right)$ at α=a.

**Figure 12:**
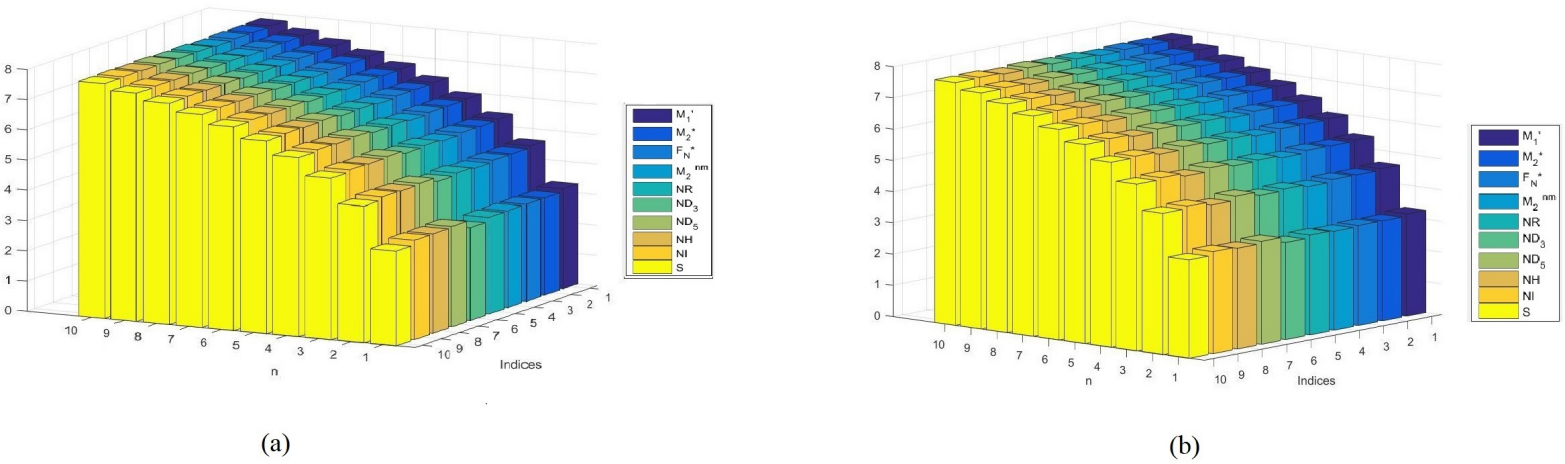
Comparison graph of neighborhood entropy measures (**a**) $VC_{5}C_{7}\left(4,4\right)$ and (**b**) $HC_{5}C_{7}\left(4,4\right)$.

This section comprises graphical interpretations and numerical tabulations of the results obtained for neighborhood degree descriptors and their corresponding entropy measures of the Pent Heptagonal Carbon Nanosheets $VC_{5}C_{7}$ and $HC_{5}C_{7}$. In addition to the comparative analysis of the nanosheets, the M-polynomial functions obtained for the structures have also been portrayed graphically. The computed numerical values for the NM polynomial fuction of the carbon sheets with its corresponding graph can be seen in Table 7 and Figure 8 respectively. The graphical comparison of various neighborhood degree indices and their corresponding entropy measures can be seen in Figure 9, Figure 10, Figure 11 and Figure 12 and their corresposding tabular representation can be seen in Table 8, Table 9, Table 10, Table 11 and Table 12 respectively.

## 7. Conclusions

Carbon nanosheets come in a broad range of structural configurations, which has facilitated the development of the material for a number of purposes, including pharmaceutical research, clinical diagnosis, vulnerability assessments and regulatory considerations. Excellent characteristics of carbon nanosheets include strong bonding, high efficiency and high stability. It is necessary to analyze chemical graphs and networks using topological descriptors in order to further investigate the fundamental topology. To obtain the vertex degree-based topological index values of the molecular structure, the M-polynomial approach has updated the algorithm. For both the nanosheets, we obtained the neighborhood degree M-polynomial function and applied the function further to evaluate various descriptors. Moreover, we have made comparison graphs for different neighborhood degree descriptors and their accompanying entropy values, as well as plotted graphs for the polynomial functions. The vertically oriented pentagonal nanostructure possesses somewhat higher numerical values than the horizontal one in the overall comparison of the various chemical attributes. In addition to the formulations, the predictive ability of these descriptors has also been studied by establishing linear regression models for some properties of the molecule. Moreover, by employing these linear models, we are able to accurately anticipate the characteristics of all other graphene derivatives.

## Figures and Tables

**Table 1:** A few of the neighborhood degree-based descriptors.

Topological Index	f(x,y)	Derivation from NM(Γ)
$M_{1}^{\prime }$	x+y	$\left(D_{x}+D_{y}\right)\left(NM\left(\Gamma \right)\right){|}_{x=y=1}$
$M_{2}^{*}$	xy	$\left(D_{x}D_{y}\right)\left(NM\left(\Gamma \right)\right){|}_{x=y=1}$
$F_{N}^{*}$	$x^{2}+y^{2}$	$\left(D_{x}^{2}+D_{y}^{2}\right)\left(NM\left(\Gamma \right)\right){|}_{x=y=1}$
$M_{2}^{nm}$	$\frac{1}{xy}$	$\left(S_{x}S_{y}\right)\left(NM\left(\Gamma \right)\right){|}_{x=y=1}$
$NR_{\alpha }$	${\left(xy\right)}^{\alpha }$	$\left(D_{x}^{\alpha }+D_{y}^{\alpha }\right)\left(NM\left(\Gamma \right)\right){|}_{x=y=1}$
$ND_{3}$	xy(x+y)	$D_{x}D_{y}\left(D_{x}+D_{y}\right)\left(NM\left(\Gamma \right)\right){|}_{x=y=1}$
$ND_{5}$	$\frac{x^{2}+y^{2}}{xy}$	$\left(D_{x}S_{y}+S_{x}D_{y}\right)\left(NM\left(\Gamma \right)\right){|}_{x=y=1}$
NH	$\frac{2}{x+y}$	$2S_{x}{J\left(NM\left(\Gamma \right)\right)|}_{x=y=1}$
NI	$\frac{xy}{x+y}$	$S_{x}JD_{x}D_{y}{\left(NM\left(\Gamma \right)\right)|}_{x=y=1}$
*S*	${\left(\frac{xy}{x+y-2}\right)}^{3}$	$S_{x}^{3}Q_{-2}JD_{x}^{3}D_{y}^{3}{\left(NM\left(\Gamma \right)\right)|}_{x=y=1}$

**Table 2:** Edge partition of $VC_{5}C_{7}\left[a,b\right]$.

Edge Partition (i,j)	Number of Edges $m_{\left(i,j\right)}$	Edge Set
(4,5)	8	$\chi_{\left\{4,5\right\}}$
(5,5)	2b+2a−4	$\chi_{\left\{5,5\right\}}$
(5,7)	2b+4a+2	$\chi_{\left\{5,7\right\}}$
(5,8)	2b−2	$\chi_{\left\{5,8\right\}}$
(6,7)	6b+4a−8	$\chi_{\left\{6,7\right\}}$
(7,9)	4b+4a−3	$\chi_{\left\{7,9\right\}}$
(8,8)	b−1	$\chi_{\left\{8,8\right\}}$
(8,9)	2b−2	$\chi_{\left\{8,9\right\}}$
(9,9)	24ab−15b−14a+10	$\chi_{\left\{9,9\right\}}$

**Table 3:** Edge partition of $HC_{5}C_{7}\left[a,b\right]$.

Edge Partition (i,j)	Number of Edges $m_{\left(i,j\right)}$	Edge Set
(4,4)	*b*	$\chi_{\left\{4,4\right\}}^{\prime }$
(4,5)	2b+4	$\chi_{\left\{4,5\right\}}^{\prime }$
(5,5)	2a−2	$\chi_{\left\{5,5\right\}}^{\prime }$
(5,7)	2	$\chi_{\left\{5,7\right\}}^{\prime }$
(5,8)	2b+4a−2	$\chi_{\left\{5,8\right\}}^{\prime }$
(6,7)	2b	$\chi_{\left\{6,7\right\}}^{\prime }$
(6,8)	2b+4a−4	$\chi_{\left\{6,8\right\}}^{\prime }$
(7,9)	b+1	$\chi_{\left\{7,9\right\}}^{\prime }$
(8,8)	b+4a	$\chi_{\left\{8,8\right\}}^{\prime }$
(8,9)	6b+8a−12	$\chi_{\left\{8,9\right\}}^{\prime }$
(9,9)	24ab−14b−22a+13	$\chi_{\left\{9,9\right\}}^{\prime }$

**Table 4:** Experimental Results for Young’s Modulus and Poisson’s Ratio of Graphene Derivatives.

Sl No.	Name	Young’s Modulus (N/m)	Poisson’s Ratio
1	Graphene	348	0.456
2	Penta Graphene	277.99	−0.078
3	Phagraphene	292.92	0.255
4	α graphyne	21.98	0.87
5	β graphyne	73.07	0.67
6	γ graphyne	165.51	0.42
7	Penta heptite graphene	292.26	0.253

**Table 5:** Computed numerical values of neighborhood descriptors for the base structures of graphene allotropes.

Sl No.	Name	NI	NH	$M_{1}^{\prime }$	$M_{2}^{\mathrm{nm}}$	${\mathrm{NR}}_{1}$	${\mathrm{ND}}_{3}$	${\mathrm{ND}}_{5}$
1	Graphene	100.63	4.6167	408	0.75217	1434	21,336	61.752
2	Penta Graphene	74.495	2.719	300	0.37919	1140	17,790	40.556
3	Phagraphene	115.52	5.1605	468	0.82801	1662	24,894	69.863
4	α Graphyne	214.85	19.112	864	4.155	2100	20,880	181.8
5	β Graphyne	168.67	13.224	684	2.7732	1812	20,160	135.34
6	γ Graphyne	145.22	10.312	588	2.0587	1650	19,632	110.55
7	Pentaheptite Graphene	89.76	4.6	364	0.81728	1244	18,222	57.679

**Table 6:** Correlation table for the properties for various neighborhood degree descriptors.

Sl No.	Descriptors	Young’s Modulus (N/m)	Poisson’s Ratio
1	*NI*	−0.918698	0.916982
2	*NH*	−0.948272	0.895635
3	$M^{\prime }{}_{1}$	−0.918348	0.918611
4	$M{{}_{2}}^{nm}$	−0.952106	0.889633
5	$NR{}_{1}$	−0.803185	0.909173
6	$ND{}_{3}$	0.214892	0.185452
7	$ND{}_{5}$	−0.940875	0.907696

**Table 7:** Computed numerical values for the *NM* polynomial function.

Sl No.	(x,y)	${\mathrm{VC}}_{5}C_{7}\left(4,4\right)$	${\mathrm{HC}}_{5}C_{7}\left(4,4\right)$
1	(−3,−3)	108,259,334,352	93,460,421,484
2	(−2,−2)	73,990,144	62,284,800
3	(−1,−1)	296	224
4	(0,0)	0	0
5	(1,1)	400	396
6	(2,2)	76,193,792	74,322,944
7	(3,3)	109,930,499,784	104,920,887,600

**Table 8:** Computed numerical values for the neighborhood indices of $VC_{5}C_{7}\left(a,a\right)$.

*a*	$M_{1}^{\prime }$	$M_{2}^{*}$	$F_{N}^{*}$	*S*	${\mathrm{ND}}_{3}$	$M_{2}^{\mathrm{nm}}$	NH	${\mathrm{ND}}_{5}$	NI
1	364	1244	2550	1745.272433	18,222	0.817283951	4.60	57.67936508	89.76014957
2	1584	6249	12,646	9402.205206	102,472	2.138058862	14.38	211.5888889	393.1922763
3	3668	15,142	30,518	23,286.9622	256,706	4.051426367	29.50	461.4984127	912.624403
4	6616	27,923	56,166	43,399.54341	480,924	6.557386464	49.95	807.4079365	1648.05653
5	10,428	44,592	89,590	69,739.94884	775,126	9.655939153	75.74	1249.31746	2599.488656
6	15,104	65,149	130,790	102,308.1785	1,139,312	13.34708444	106.85	1787.226984	3766.920783
7	20,644	89,594	179,766	141,104.2324	1,573,482	17.63082231	143.30	2421.136508	5150.35291
8	27,048	117,927	236,518	186,128.1104	2,077,636	22.50715278	185.09	3151.046032	6749.785036
9	34,316	150,148	301,046	237,379.8127	2,651,774	27.97607584	232.21	3976.955556	8565.217163
10	42,448	186,257	373,350	294,859.3393	3,295,896	34.03759149	284.66	4898.865079	10,596.64929

**Table 9:** Computed numerical values for the neighborhood indices of $HC_{5}C_{7}\left(a,a\right)$.

*a*	$M_{1}^{\prime }$	$M_{2}^{*}$	$F_{N}^{*}$	*S*	${\mathrm{ND}}_{3}$	$M_{2}^{\mathrm{nm}}$	NH	${\mathrm{ND}}_{5}$	NI
1	356	1217	2504	1687.166041	17,606	0.75892306	4.346863104	55.79761905	87.63862853
2	1572	6242	12,654	9385.591899	102,590	2.056929012	13.92627846	208.0531746	389.8091961
3	3652	15,155	30,580	23,311.84198	257,558	3.947527557	28.83902715	456.3087302	907.9797637
4	6596	27,956	56,282	43,465.91627	482,510	6.430718695	49.08510917	800.5642857	1642.150331
5	10,404	44,645	89,760	69,847.81478	777,446	9.506502425	74.66452453	1240.819841	2592.320899
6	15,076	65,222	131,014	102,457.5375	1,142,366	13.17487875	105.5772732	1777.075397	3758.491466
7	20,612	89,687	180,044	141,295.0845	1,577,270	17.43584766	141.8233552	2409.330952	5140.662034
8	27,012	118,040	236,850	186,360.4556	2,082,158	22.28940917	183.4027706	3137.586508	6738.832602
9	34,276	150,281	301,432	237,653.651	2,657,030	27.73556327	230.3155193	3961.842063	8553.003169
10	42,404	186,410	373,790	295,174.6706	3,301,886	33.77430996	282.5616013	4882.097619	10,583.17374

**Table 10:** Computed numerical values for $NR_{\alpha }\left(VC_{5}C_{7}\left(a,a\right)\right)$ and $NR_{\alpha }\left(HC_{5}C_{7}\left(a,a\right)\right)$.

*a*	${\mathrm{NR}}_{\alpha }\left({\mathrm{VC}}_{5}C_{7}\left(a,a\right)\right)$	${\mathrm{NR}}_{\alpha }\left({\mathrm{HC}}_{5}C_{7}\left(a,a\right)\right)$
1	1244	1217
2	428,207	427,468
3	84,071,002	83,691,503
4	12,795,317,113	12,682,488,234
5	1,686,019,988,652	1,665,833,260,265
6	202,652,609,771,763	199,819,539,312,344
7	22,862,655,764,319,500	22,515,947,309,170,400
8	2,462,993,185,867,150,000	2,424,074,735,575,690,000
9	256,207,126,287,300,000,000	252,088,189,460,165,000,000
10	25,929,791,678,574,900,000,000	25,511,980,566,925,000,000,000

**Table 11:** Computed numerical values for neighborhood degree based entropy measures of $VC_{5}C_{7}$.

*a*	$M_{1}^{\prime }$	$M_{2}^{*}$	$F_{N}^{*}$	$M_{2}^{\mathrm{nm}}$	${\mathrm{NR}}_{\alpha }$	${\mathrm{ND}}_{3}$	${\mathrm{ND}}_{5}$	NH	NI	*S*
1	3.2999	3.208387743	3.214	3.2108	3.2084	3.0813	3.332	3.2999	3.2987	3.14640049
2	4.6219	4.566216859	4.5707	4.5159	4.5662	4.4987	4.6441	4.6033	4.6206	4.528317821
3	5.412849284	5.373599659	5.3769	5.3178	5.3736	5.328	5.4291	5.3941	5.4118	5.347048954
4	5.97849477	5.948309958	5.9509	5.8953	5.9483	5.9139	5.9913	5.9617	5.9776	5.927965587
5	6.419052436	6.39455544	6.3967	6.3459	6.3946	6.367	6.4295	6.4041	6.4183	6.378086177
6	6.779919258	6.759314635	6.7611	6.7149	6.7593	6.7363	6.7888	6.7666	6.7793	6.745487223
7	7.085544767	7.067768558	7.0693	7.0272	7.0678	7.048	7.0933	7.0736	7.085	7.05585535
8	7.350612522	7.334983534	7.3364	7.2977	7.335	7.3177	7.3574	7.3397	7.3501	7.324520276
9	7.584633973	7.57069021	7.5719	7.5363	7.5707	7.5553	7.5907	7.5747	7.5842	7.56136292
10	7.794123783	7.78153766	7.7827	7.7496	7.7815	7.7677	7.7997	7.785	7.7937	7.77312421

**Table 12:** Computed numerical values for neighborhood degree based entropy measures of $HC_{5}C_{7}$.

*a*	$M_{1}^{\prime }$	$M_{2}^{*}$	$F_{N}^{*}$	$M_{2}^{\mathrm{nm}}$	${\mathrm{NR}}_{\alpha }$	${\mathrm{ND}}_{3}$	${\mathrm{ND}}_{5}$	NH	NI	*S*
1	3.2688	3.1985	3.2034	3.167	3.1985	3.1078	3.2952	3.2247	3.2677	3.1538
2	4.6045	4.5562	4.5613	4.4811	4.5562	4.5012	4.6244	4.4966	4.6029	4.5238
3	5.4009	5.3667	5.3705	5.2892	5.3667	5.3292	5.4157	5.3026	5.3997	5.3438
4	5.9695	5.9431	5.9462	5.8712	5.9431	5.9148	5.9811	5.8844	5.9685	5.9255
5	6.4118	6.3904	6.3929	6.3251	6.3904	6.3677	6.4213	6.3379	6.411	6.3762
6	6.7739	6.7559	6.758	6.6967	6.7559	6.7369	6.7819	6.7089	6.7731	6.7439
7	7.0804	7.0648	7.0667	7.0109	7.0648	7.0486	7.0874	7.0225	7.0797	7.0545
8	7.3461	7.3324	7.334	7.283	7.3324	7.3182	7.3522	7.294	7.3455	7.3234
9	7.5806	7.5684	7.5699	7.5229	7.5684	7.5558	7.5861	7.5333	7.5801	7.5603
10	7.7905	7.7795	7.7808	7.7374	7.7795	7.7681	7.7955	7.7472	7.7900	7.7722

## Data Availability

The data used to support the study are cited within the text as references.

## References

[1] Gao W., Wang W., Farahani M.R. (2016). Topological indices study of molecular structure in anticancer drugs. J. Chem..

[2] Nadeem M.F., Azeem M., Farman I. (2022). Comparative study of topological indices for capped and uncapped carbon nanotubes. Polycycl. Aromat. Compd..

[3] Fan H., Shen W. (2015). Carbon nanosheets: Synthesis and application. ChemSusChem.

[4] Edwards S.L., Werkmeister J.A., Ramshaw J.A. (2009). Carbon nanotubes in scaffolds for tissue engineering. Expert Rev. Med. Devices.

[5] Saito N., Usui Y., Aoki K., Narita N., Shimizu M., Hara K., Ogiwara N., Nakamura K., Ishigaki N., Kato H. (2009). Carbon nanotubes: Biomaterial applications. Chem. Soc. Rev..

[6] Ghani M.U., Campena F.J.H., Ali S., Dehraj S., Cancan M., Alharbi F.M., Galal A.M. (2023). Characterizations of Chemical Networks Entropies by K-Banhatii Topological Indices. Symmetry.

[7] Liu J.B., Pan X.F. (2016). Minimizing Kirchhoff index among graphs with a given vertex bipartiteness. Appl. Math. Comput..

[8] Ghani M.U., Campena F.J.H., Maqbool M.K., Liu J.B., Dehraj S., Cancan M., Alharbi F.M. (2023). Entropy Related to K-Banhatti Indices via Valency Based on the Presence of C_6_H_6_ in Various Molecules. Molecules.

[9] Liu J.B., Zhao J., Min J., Cao J. (2019). The Hosoya index of graphs formed by a fractal graph. Fractals.

[10] Tag El Din E.S.M., Sultan F., Ghani M.U., Liu J.B., Dehraj S., Cancan M., Alharbi F.M., Alhushaybari A. (2022). Some Novel Results Involving Prototypical Computation of Zagreb Polynomials and Indices for SiO_4_ Embedded in a Chain of Silicates. Molecules.

[11] Mondal S., Siddiqui M.K., De N., Pal A. (2021). Neighborhood M-polynomial of crystallographic structures. Biointerface Res. Appl. Chem..

[12] Haoer R.S. (2021). Topological indices of metal-organic networks via neighborhood M-polynomial. J. Discret. Math. Sci. Cryptogr..

[13] Ghani M.U., Kashif Maqbool M., George R., Ofem A.E., Cancan M. (2022). Entropies via Various Molecular Descriptors of Layer Structure of H_3_BO_3_. Mathematics.

[14] Liu J.B., Wang C., Wang S., Wei B. (2019). Zagreb indices and multiplicative zagreb indices of eulerian graphs. Bull. Malays. Math. Sci. Soc..

[15] Zhang Y.F., Ghani M.U., Sultan F., Inc M., Cancan M. (2022). Connecting SiO_4_ in Silicate and Silicate Chain Networks to Compute Kulli Temperature Indices. Molecules.

[16] Liu J.B., Zhao J., He H., Shao Z. (2019). Valency-based topological descriptors and structural property of the generalized sierpiński networks. J. Stat. Phys..

[17] Nagarajan S., Imran M., Kumar P.M., Pattabiraman K., Ghani M.U. (2023). Degree-Based Entropy of Some Classes of Networks. Mathematics.

[18] Rajpoot A., Selvaganesh L. (2021). Extension of M-polynomial and degree based topological indices for nanotube. TWMS J. Appl. Eng. Math..

[19] Chaudhry F., Shoukat I., Afzal D., Park C., Cancan M., Farahani M.R. (2021). M-polynomials and degree-based topological indices of the molecule copper (I) oxide. J. Chem..

[20] Ghani M.U., Campena F.J.H., Pattabiraman K., Ismail R., Karamti H., Husin M.N. (2023). Valency-Based Indices for Some Succinct Drugs by Using M-Polynomial. Symmetry.

[21] Ali S., Ismail R., Campena F.J.H., Karamti H., Ghani M.U. (2023). On Rotationally Symmetrical Planar Networks and Their Local Fractional Metric Dimension. Symmetry.

[22] Guangyu L., Hussain S., Khalid A., Ishtiaq M., Siddiqui M.K., Cancan M., Imran M. (2022). Topological Study of Carbon Nanotube and Polycyclic Aromatic Nanostar Molecular Structures. Polycycl. Aromat. Compd..

[23] Ullah A., Qasim M., Zaman S., Khan A. (2022). Computational and comparative aspects of two carbon nanosheets with respect to some novel topological indices. Ain Shams Eng. J..

[24] Deng F., Zhang X., Alaeiyan M., Mehboob A., Farahani M.R. (2019). Topological indices of the pent-heptagonal nanosheets VC_5_C_7_ and HC_5_C_7_. Adv. Mater. Sci. Eng..

[25] Munir M.M. (2022). Irregularity molecular descriptors of VC_5_C_7_ [m, n] and HC_5_C_7_ [m, n] nanotubes. Front. Phys..

[26] Farahani M.R. (2013). Connectivity indices of pent-heptagonal nanotubes. Adv. Mater. Corros..

[27] Ishtiaq M., Rauf A., Rubbab Q., Siddiqui M.K., Rehman A.U., Cancan M. (2022). A Degree Based Topological Study of Two Carbon Nanosheets VC_5_C_7_ and HC_5_C_7_. Polycycl. Aromat. Compd..

[28] Mondal S., Dey A., De N., Pal A. (2021). QSPR analysis of some novel neighborhood degree-based topological descriptors. Complex Intell. Syst..

[29] Ghani M.U., Sultan F., Tag El Din E.S.M., Khan A.R., Liu J.B., Cancan M. (2022). A Paradigmatic Approach to Find the Valency-Based K-Banhatti and Redefined Zagreb Entropy for Niobium Oxide and a Metal–Organic Framework. Molecules.

[30] Chu Y.M., Khan A.R., Ghani M.U., Ghaffar A., Inc M. (2022). Computation of zagreb polynomials and zagreb indices for benzenoid triangular & hourglass system. Polycycl. Aromat. Compd..

[31] Alam A., Ghani M.U., Kamran M., Shazib Hameed M., Hussain Khan R., Baig A.Q. (2022). Degree-Based Entropy for a Non-Kekulean Benzenoid Graph. J. Math..

[32] Sarkar P., De N., Pal A. (2022). On some topological indices and their importance in chemical sciences: A comparative study. Eur. Phys. J. Plus.

[33] Kier L.B., Hall L.H. (1990). An electrotopological-state index for atoms in molecules. Pharm. Res..

[34] Hall L.H., Mohney B., Kier L.B. (1991). The electrotopological state: An atom index for QSAR. Quant. Struct. Relatsh..

[35] Bender A., Glen R.C. (2005). A discussion of measures of enrichment in virtual screening: Comparing the information content of descriptors with increasing levels of sophistication. J. Chem. Inf. Model..

[36] Randic M. (1975). Characterization of molecular branching. J. Am. Chem. Soc..

[37] Kier L.B., Hall L.H. (1986). Molecular Connectivity in Structure-Activity Analysis.

[38] Mondal S., De N., Pal A. (2019). On some new neighborhood degree based indices. arXiv.

[39] Li X., Shi Y. (2008). A survey on the Randic index. MATCH Commun. Math. Comput. Chem..

[40] Hao J. (2011). Theorems about Zagreb indices and modified Zagreb indices. MATCH Commun. Math. Comput. Chem..

[41] Hosamani S.M. (2017). Computing Sanskruti index of certain nanostructures. J. Appl. Math. Comput..

[42] Sabirov D.S., Shepelevich I.S. (2021). Information entropy in chemistry: An overview. Entropy.

[43] Rahul M., Clement J., Junias J.S., Arockiaraj M., Balasubramanian K. (2022). Degree-based entropies of graphene, graphyne and graphdiyne using Shannon’s approach. J. Mol. Struct..

[44] Yang J., Konsalraj J., Raja S A.A. (2023). Sum Degree-Based Indices and Entropy Measures for Certain Family of Graphene Molecules. Molecules.

[45] Adnan M., Bokhary S.A.U.H., Abbas G., Iqbal T. (2022). Degree-based topological indices and QSPR analysis of antituberculosis drugs. J. Chem..

[46] Consonni V., Todeschini R. (2009). Molecular Descriptors for Chemoinformatics: Volume I: Alphabetical Listing.

[47] Hosamani S., Perigidad D., Jamagoud S., Maled Y., Gavade S. (2017). QSPR analysis of certain degree based topological indices. J. Stat. Appl. Probab..

[48] Crespi V.H., Benedict L.X., Cohen M.L., Louie S.G. (1996). Prediction of a pure-carbon planar covalent metal. Phys. Rev. B.

[49] Li X., Li B.H., He Y.B., Kang F.Y. (2020). A review of graphynes: Properties, applications and synthesis. New Carbon Mater..

[50] Enyashin A.N., Ivanovskii A.L. (2011). Graphene allotropes. New Carbon Mater. Phys. Status Solidi B.

[51] Puigdollers A.R., Alonso G., Gamallo P. (2016). First-principles study of structural, elastic and electronic properties of *α*-, *β*- and *γ*-graphyne. Carbon.

[52] Sun H., Mukherjee S., Singh C.V. (2016). Mechanical properties of monolayer penta-graphene and phagraphene: A first-principles study. Phys. Chem. Chem. Phys..

[53] Zhou L., Wang Y., Cao G. (2013). Elastic properties of monolayer graphene with different chiralities. J. Phys. Condens. Matter.

